# Toward a radically embodied neuroscience of attachment and relationships

**DOI:** 10.3389/fnhum.2015.00266

**Published:** 2015-05-21

**Authors:** Lane Beckes, Hans IJzerman, Mattie Tops

**Affiliations:** ^1^Department of Psychology, Bradley University, PeoriaIL, USA; ^2^Department of Clinical Psychology, VU University AmsterdamAmsterdam, Netherlands; ^3^Tilburg School of Behavioral and Social Sciences, Tilburg UniversityTilburg, Netherlands

**Keywords:** attachment, embodied cognition, interpersonal relationships, thermoregulation, neurobiology, oxytocin, ecological psychology

## Abstract

Attachment theory (Bowlby, 1969/1982) posits the existence of internal working models as a foundational feature of human bonds. Radical embodied approaches instead suggest that cognition requires no computation or representation, favoring a cognition situated in a body in an environmental context with affordances for action (Chemero, 2009; Barrett, 2011; Wilson and Golonka, 2013; Casasanto and Lupyan, 2015). We explore whether embodied approaches to social soothing, interpersonal warmth, separation distress, and support seeking could replace representational constructs such as internal working models with a view of relationship cognition anchored in the resources afforded to the individual by their brain, body, and environment in interaction. We review the neurobiological bases for social attachments and relationships and attempt to delineate how these systems overlap or don’t with more basic physiological systems in ways that support or contradict a radical embodied explanation. We suggest that many effects might be the result of the fact that relationship cognition depends on and emerges out of the action of neural systems that regulate several clearly physically grounded systems. For example, the neuropeptide oxytocin appears to be central to attachment and pair-bond behavior (Carter and Keverne, 2002) and is implicated in social thermoregulation more broadly, being necessary for maintaining a warm body temperature (for a review, see IJzerman et al., 2015b). Finally, we discuss the most challenging issues around taking a radically embodied perspective on social relationships. We find the most crucial challenge in individual differences in support seeking and responses to social contact, which have long been thought to be a function of representational structures in the mind (e.g., Baldwin, 1995). Together we entertain the thought to explain such individual differences without mediating representations or computations, but in the end propose a hybrid model of radical embodiment and internal representations.

## Toward a Radically Embodied Neuroscience of Attachment and Close Relationships?

People’s most intimate connections are bound to their earliest social interactions. These have been suggested to lead to *internal working models* of people’s social world. Or so attachment theory (Bowlby, 1969/1982; see also Craik, 1943) has suggested. Highly influential, innovative, and integrative, the theory has grown to be one of the most generative theories of interpersonal relationships in psychology and human development. Accordingly it has provided a basis for strong claims about the nature of human relationships and human cognition. Some of those claims, such as the claim that humans are innately social animals, and that being social has consequences for mental and physical well-being, are nigh indisputable given the current support (e.g., Baumeister and Leary, 1995; Holt-Lunstad et al., 2010; Beckes and Coan, 2011).

Other claims, however, such as the idea that people develop internal working models of their relationships and that those models influence behavior from cradle to grave are more debatable and our understanding of the processes that lead up the formation of such internal working models are still in their rudimentary phases. Many relationship theories that spring from attachment theories either explicitly posit or imply representational schemata or computational thought (e.g., Baldwin, 1992; Agnew et al., 1998; Andersen and Chen, 2002; Simpson, 2007). Here we entertain the thought that, in many instances, the cognitive neuroscience and psychology of relationships does not require representational cognition by “putting brain, body, and social relationships together again” (cf. Clark, 1998; see also Hendriks-Jansen, 1996). Notably, our primary goal with this article is not to argue that *all* attachment processes are necessarily radically embodied, but rather to present support for the radical view, taking this possibility as far as we can. We thus make the case that radically embodied approaches have the potential to benefit the study of attachment processes through the presentation of considerable research on the neurobiological side of attachment behavior that fits well within that framework. Further, we wish to make the case that the most fruitful approach to attachment might be one that includes both non-representational approaches *and* representational approaches. We end the article by framing the brain as having evolved in such a way that the representational architecture of the brain is *scaffolded* “on top of” non-representational cognition, which should thus be situated to a greater degree, and also constrained by environmental stimuli to a greater degree.

To examine both normative and individual differences’ facets of relationship psychology from a radically embodied cognitive neuroscience perspective we will review empirical findings from both animal models and human investigations of interpersonal behavior and neurobiology. Our goal is to discover the “cognitive architecture” for attachment starting with non-representational approaches such as Gibsonian (Gibson, 1979) ecological psychology and traditional behaviorist learning approaches (e.g., Skinner, 1953). We will briefly define how we interpret the non-representational cognition and radically embodied cognitive neuroscience perspectives (see, Chemero, 2009; Barrett, 2011; Wilson and Golonka, 2013) and framing this from an attachment perspective (Bowlby, 1969/1982) and its modern perspectives (e.g., Hazan and Shaver, 1987; Mikulincer and Shaver, 2003).

Then, we will discuss support for the idea that the body – and its corresponding neural activations – plays a crucial part in interpersonal interactions, leaning heavily on the animal literature to describe sensory pathways through which social interaction influences psychological and physiological functioning (e.g., Hofer, 2006), and tie this literature with the sparser work in the human neurosciences related to interpersonal processes (e.g., Coan et al., 2006). We will suggest that many attachment phenomena can be understood as a dynamic coupling of the organism to its environment in which researchers’ interpretation via representational processes may be unnecessary (and perhaps even incorrect), simply relying on the organism’s homeostatic process. In order to take this radical embodiment view as far as we can, we will entertain the idea that many neural processes relating to individually variant attachment styles can be understood through their ties with the body. Subsequently we will reveal fundamental links between bodily states and relationship cognitions (e.g., IJzerman and Semin, 2009, 2010) with a discussion – and a framing through a radical embodiment lens – on work done on what some may term conceptual embodiment. In so doing, we will discuss Zajonc and Markus’ (1984) dichotomy between soft and hard interfaces of cognition, but will also suggest the theory of predictive and reactive control systems (PARCSs; Tops et al., 2010) to elucidate how representational cognition may emerge from more basic, radically embodied cognitive systems, providing an integration of non-representational approaches and representational approaches at the neural level. In the end, we will suggest a research agenda that might falsify positions we set forth here.

## Basic Principles of Radically Embodied Cognition

The primary goal of radically embodied cognitive perspectives is to understand various psychological processes in the most parsimonious way possible (in this case questioning whether the mediation of any additional representational mechanisms are obsolete; Chemero, 2009; Barrett, 2011; Wilson and Golonka, 2013). Chemero (2009) offers one approach to this problem by offering some key identifiers of radical embodiment versus representation. Chemero’s approach is eliminitavist in nature (cf. Fodor and Pylyshyn, 1988), rooted in the American functionalism of James (1884). A contrasting strain of thought is representationalism, rooted in the German structuralist school of Wilhelm Wundt. One major component that differs between the two is whether the mind is composed of individual parts that represent the world. Chemero (2009) identifies the representionalist position as involving internal representations upon which people perform *mental gymnastics*. Eliminitavists wonder whether the mind’s, and therefore cognition’s, primary task is something entirely different from “construction, manipulation, and use of representations of the world” (Chemero, 2009, p. 18). In this sense, radical embodiment can be understood primarily through its rejection of mediating representations, and its embrace of the idea that explanations should look to the dynamic coupling of information in the environment, sensory experience, body features, and opportunities for behavior. Moreover, once all constraints within this system are known, those constraints provide sufficient information to guide action, without the need for complex calculation.

Skinner’s (1953) brand of behaviorism is perhaps the most well-known eliminitavist approach. Skinner took a relatively agnostic position in regards to the nature of representations, instead focusing on processes that were easily observable and measurable. Following the cognitive revolution, Gibson’s (1979) ecological psychology carried the eliminitavist banner. Chemero (2009) notes that Gibson proposed three major ideas. First, perception is direct and internal information need not be computed or represented to go from sensation to perception. In this sense external stimuli are *presented*, not re-presented (like a radio presents the light information from radio waves as sounds created by a vibrating speaker cone). External sensations may change form, as in transduction, but the information contained in the external agent is the same as the information presented in the perceiver’s nervous system.

Second, similar to James’ (1884) view on action, perception should be for doing, as perception primarily serves the functional act of achieving behavior. Thus, people viewing their partner as sad may notice (consciously or not) changes in their sympathetic nervous system, which moves them to act to sooth their partner. Third, because perception should be direct and because it is able to produce functional behavior, the environment must contain all necessary information for an organism to engage in adaptive behavior (cf. Griffiths and Scarantino, 2009). More specifically, Gibson (1979) suggested that people directly perceive affordances of action opportunities, like soothing or retaliating against another, present in the current environment in a matter specified by the direct information in the environment. This approach allows the organism to collect information rich sensory input and use that information to guide action in a manner that does not require the mediation of representational constructs or computations. In this sense perception is primarily of information, which can be used to perceive affordances available in the current environment.

But how does this work in interpersonal relationships? We will attempt to discuss the radically embodied cognitive neuroscience of attachment and relationship processes primarily by specifying the biological and environmental stimuli that constrain those processes. Further, by adopting the premises of ecological psychology and behaviorism, we will entertain the thought of radically embodied mechanisms for explaining attachment relationships. We believe that these approaches could be fruitful for understanding relationship processes regardless of the “true” nature of the human mind, in particular shifting from the typical self-focused nature of social psychology to a more dynamic relationship-focused nature of cognitive systems.

## Starting Assumptions of Attachment Theory

Attachment theory proposes that relationships serve functions that solve problems related to evolutionary pressures. In attachment theory, an “attachment system” is the primary motivator for the bonds that tie children to their parents. The term “attachment system” is a bit of a misnomer, because Bowlby (1969/1982) described instead the coordination and action of multiple instinctive systems. The primary proximal function of each instinct is the maintenance of proximity to the child’s primary caregiver. What begins as individual instinctive behaviors with the same general function, over time (i.e., by 18 months of age) get coordinated into “sophisticated goal-corrected systems” (Bowlby, 1969/1982, p. 180), which effectively coordinate behavior to reach the desired end state. Hallmark behaviors include suckling, clinging, crying, smiling, and following, all in the service of drawing the caregiver closer to provide warmth and security. This keeps the child safe from predation and isolation, which holds with it the risk of starvation and injury. In addition to such survival benefits, proximity maintenance also seems to fulfill a secondary function of affect and physiological regulation, which may promote exploration and affiliation, leading to better reproductive fitness (see Zeifman and Hazan, 2008).

In children, the attachment system is activated by distressing circumstances (Bowlby, 1969/1982), including separation from caregivers, illness, injury, pain, and fear (e.g., due to the presence of a predator). The activation of the system motivates proximity seeking or maintaining behavior. Generally the system is deactivated, and attachment behaviors are terminated, through sight, sound, touch, and other forms of perceptual contact with the caregiver. Once calmed, the child resumes exploratory behavior until separation or another distressing event. Thus, attachment theory at its core is about the very immediate, and innately motivated management of proximity to caregivers on the one hand, and exploration of the environment on the other.

In the last 30 years scholars have made serious attempts to extend attachment theory to adult relationships. Seminally, Hazan and Shaver (1987) asserted that the attachment system is partially responsible for the adult romantic bond. Indeed, multiple parallels have been drawn between behavior in infant-caregiver interactions and adult romantic partner interactions. Zeifman and Hazan (2008) offer a fairly extensive account noting the similarities in coordinative systems (such as nuzzling, kissing, suckling, ventral–ventral contact, and mutual eye gaze) and stages of separation (i.e., protest, despair, and detachment; Bowlby, 1980; Hazan and Shaver, 1992). We continue now into a representational approach, and reinterpret these findings to frame them with a departure point from the radical view.

## Is Attachment Radically Embodied?

In the past decades, social psychology has seen a revival in studying the importance of the body in a host of different processes. These processes have been studied from a perspective relying on *soft* and *hard* representations of the external environment. Such hard representations are the direct consequence of the stimulus in the external environment, while soft representations are postulated as cognitively mediated or appraisal processes that cause the phenomenological experience related to the external stimulus, without this stimulus being present. In regards to attachment processes, in humans there is growing support relating temperature cues to close relationships. Often, these effects are related to what one may call “conceptual embodiment,” of which Lakoff and Johnson’s (1999) account of conceptual metaphors is a typical example (see also Landau et al., 2010). In brief, the conceptual metaphor view postulates that people co-experience an abstract concept and concrete experience, and from thereon form internal models that combine the aspect of experience and concept to imbue meaning to their social world (an idea that was antedated by Asch, 1958, early writings).

A compelling possibility regarding the embodiment of attachment and relationship processes is the potential that relationship cognition is fundamentally linked to the body’s regulation of body temperature. In contrast to Lakoff and Johnson’s (1999) conceptual embodiment account, IJzerman and Koole (2011) suggested that conceptual metaphors that invoke physical descriptions for non-physical ideas might not have a representational structure as normally conceived in cognitive science, and instead rely on prepared physiological processes. Although at an intuitive level one may expect that warmth and affection are provided structure through distinct areas in the brain (as postulated by metaphor theories), it is more likely that we can interpret these earlier results through the radical embodiment lens.

One admirable aspect of Lakoff and Johnson’s (1999) theory is that it includes a falsifiable prediction, by claiming that the effect of concrete experiences flow onto abstract target dimensions only (and not vice versa). Specifically, IJzerman and Koole (2011) explained that this aspect of their theory was not true for warmth related effects (see also Lee and Schwarz, 2012; for skepticism of this view), as they discuss findings showing that relationship warmth has an effect on the perception of ambient temperature (for original studies and replications, see Zhong and Leonardelli, 2008; IJzerman and Semin, 2010; Szymkow et al., 2013; IJzerman et al., 2015a; for a notable non-replication, see Ebersole et al., 2015). As an alternative to Lakoff and Johnson’s model, IJzerman and Koole (2011) identified Barsalou’s (1999, 2008) perceptual symbol systems (PSSs) as a rival candidate for attachment-related processes regarding underlying psychological processes in which concepts are built in perceptual systems through simulations relying on perceptual elements.

The PSS view may first rely on very basic social thermoregulatory mechanisms. That is, IJzerman et al. (2012) found that skin temperature might account for some of the effects that have been obtained earlier. That is, after a brief period of social exclusion, skin temperature of participants decreased (while negative affect typically so experienced after rejection could also be alleviated via a warm cup of tea). Note that the exact relationships between skin temperature and temperature perceptions have not been settled, but we do know that people with a lower skin temperature perceive drops in temperature more quickly.

Thermoregulatory accounts seem to apply more broadly and may antedate the workings of the “simulator.” That is, Fransson et al. (2005) found that babies have smaller differences between skin temperature and core temperature when being held (vs. when not) potentially preventing hypothermia. Additionally, skin-to-skin contact leads the feet of neonates (that were skin-to-skin, as compared to those that were removed from the skin of the mother and swaddled) to have a greater increase in skin temperature (Bystrova et al., 2007; for a summary see IJzerman et al., 2015b). In other words, many of the processes that may previously have been attributed to “conceptual embodiment” may well be attributable to relatively simple changes in skin temperature – a factor crucial in animals’ regulation of body temperature and something that IJzerman et al. (2012) termed an “evolved simulator.” As such, homeostatic systems may serve as embodied signals for relationship states, requiring no mediating representations. We contend that such a link between relationship cognition and sensory perception is rooted in an evolved link between energy regulation and social contact.

## The Organism’s Most Essential Goals and Needs: The Regulation of Energy

Attachment may be radically embodied because it has evolved to serve the homeostatic needs of the organism. Specifically, social animals appear to leverage social relationships as an efficient method of regulating homeostasis and energy use. Therefore, attachment may be primarily embodied to support these homeostatic systems. Before we address the issue of homeostasis, why would the regulation of homeostasis be tied to properties of the attachment system? We rely on social baseline theory (SBT; cf. Proffitt, 2006; Beckes and Coan, 2011) to provide us with a first premise that may help us answer this question.

Social baseline theory relies on two basic principles. First, metabolic resources are regulated more efficiently when done jointly. Second, the organism relies on affordances that help with regulating this “social baseline.” This latter fact is an acknowledgment that the natural human ecological niche is not a primarily physical, but instead a social niche (Brewer and Caporael, 1990; Berscheid, 2003). Humans have adapted to almost every terrestrial environment, primarily through cooperative social behavior. Further, it is now clear that human health is greatly enhanced when a person is embedded in a rich, supportive social network, and greatly diminished by the lack of such a network (Gallagher and Vella-Brodick, 2008; Beals et al., 2009; Cohen and Janicki-Deverts, 2009; Holt-Lunstad et al., 2010). Moreover, one can easily find examples of individuals that survive and even thrive without capabilities normally considered fundamental. It is relatively easy to find examples of people who have disabilities related to sensation, such as those who are blind or deaf, or movement such as those who are paraplegic or quadriplegic, and are nonetheless well-adjusted and healthy individuals. Alternatively, finding individuals who are simultaneously well-adjusted, healthy, and socially isolated is nearly impossible. But why does attachment relate to the regulation of metabolic resources?

Biology necessitates that all organisms must use less energy than they take in (cf. Proffitt, 2006), a simple principle referred to as *economy of action* (see also, Stearns, 1992; Kaplan and Gangestad, 2005). The most important categories in animals’ lives relate to survival, growth, reproduction, and rearing. The importance of each is dependent on an animal’s ecological niche and evolved strategies for that niche. The concept that energy must be distributed across these general categories of tasks seems to be a core feature of evolutionary biology. Crucially, humans appear to have developed an unique ecological niche, one that is primarily social in nature (Beckes and Coan, 2011), and requires heavy investment in offspring (typically thought of as K-selection; MacArthur and Wilson, 1967; Trivers, 1972). SBT postulates that others help regulate metabolic resources, both in dyadic relationships as well as throughout development. Throughout development, the investment in offspring requires a sharp reduction in the quantity of offspring that humans can have over a lifetime, but with that tradeoff in reproductive output comes longer lifespans and greater likelihood that any given child will survive to reproductive age and reproduce itself.

The idea that others regulate metabolic resources can be understood from SBT’s second principle, namely the Gibsonian suggestion that affordances, at their core, are for action (James, 1884). But what do we mean by affordances in the context of attachment? Chemero (2009) defines the idea of affordances as relations between a feature of the environment and an animal’s ability to act on that feature; formally: Affords-ϕ (feature, ability). In this case the attachment figure has features that might provide support (e.g., softness, body heat, the capacity to defend the individual because of numbers or size, arms and a trunk the child can cling to and wrap around), and the person’s ability is directly related to their capacity to elicit the type of support needed (e.g., eliciting soft caresses, eliciting huddling or holding behaviors, eliciting defensive aggression toward predators or competitors). This definition is useful in thinking about attachment in an embodied context because it explicitly defines an affordance as a relation, in this case between an organism and the body of another organism.

These principles are perhaps best illustrated by an empirical demonstration based on Gibsonian affordance principles. Proffitt (2006) suggests that energy dynamics influence perception through affordance processes such that activities such as climbing a hill are perceived as more difficult if one is tired, weak, or hauling a heavy backpack. First, Stefanucci et al. (2005) found that wearing a heavy backpack made an uphill incline seem steeper. This suggests the manner in which affordances modify perception of the environment in support of action. From our attachment perspective, being alone is like wearing a heavy backpack. Indeed, others help regulate metabolic resources, as Schnall et al. (2008, see also Oishi et al., 2013) found that having a friend nearby also reduced the perception of a slant of a hill, and did so in a correlated manner with the length of relationship. Thus, social proximity decreases costs of action in the environment by diminishing vigilance and freeing up metabolic and sensory resources from defensive processes for exploration, social interaction, and resource gathering.

Beyond this previous example of why being alone may change one’s perception, it is important to understand why exactly this occurs. Why do social resources scale the perception of our environment? The affordances we cite are related directly to the biological imperatives of the organism. These imperatives *require* the organism to maintain a baseline across a variety of physical and psychological resources and a crucial baseline is the social one. Gross and Proffitt (2013) reasoned that perception is scaled both by physiological and social resources, and information regarding both types of resources are compared to baselines to determine the costs and benefits of engaging in any given action. It is the – embodied – perception of information regarding physiological and social resources, along with perception of the physical situation that scales perception. If resources – including social ones – are high, then action appears easier, scaling the environment in a way that seems easier to manage. If they are low, then that scaling changes to make any given task appear more difficult.

From here we note that affordances at their most basic level are action opportunities. Moreover, they may represent opportunities for a gain of some sort, or to avoid some real or perceived danger (Sanders, 1997). Load sharing (Beckes and Coan, 2011) is the primary mechanism by which social resources are thought to alter human perceptions of the costs of engaging in any particular action. Because humans have primarily evolved to live in a social ecological niche, we assume the presence of a familiar social network, which can help to complete tasks, solve problems, and maintain homeostasis. This likely evolved initially from adaptations inducing risk distribution behavioral strategies; many animals will move in herds, flocks, or other groupings partly because the group offers a level of protection, reducing risk, and making the environment appear easier to manage. In this sense we oﬄoad problem solving and effort to the group, reducing the cost of acting for each individual member. This is because the group becomes an emergent organism with a different set of constraints and abilities.

Based on the principles of economy of action and load sharing, one can reason that individuals acting in concert, synergistically, create a new perception-action system that cannot be decomposed into its individual parts (see e.g., Marsh et al., 2006; see also Marsh et al., 2009). As Marsh et al. (2006, p. 20) note this creates a new unit of perception-action coupling. Thus, social coordination emerges out of “maximizing the patterns of information flow required for a successful social encounter.” Individuals will tend to synchronize their behavior with the others in their environment for the purposes of social interaction, relying on speech patterns, vocalizations (including pitch, prosody, etc.), movement, and touch by others in service of coordinating behavior.

Such optimization should be more efficient with attachment figures, leading to improved coordination and more efficient coupling. Think, for example, of a classic example provided by Asch (1958), who described how two boys move an obstacle that neither is capable of moving alone. He noted that they carefully synchronize their behavior and adjust for sudden movements of the other. In this case, it is more reasonable to assume they are acting with a shared set of affordances, with perception-action coupling linked to one another, and constrained by their combined abilities. In other words, close coordination of attachment relations should be easily explained through non-representational means via the features of normative attachment in terms of sensory stimuli, environmental information, and affordances.

Beyond these relatively more complex social interactions, others provide humans with a unique set of elementary affordances (Marsh et al., 2009). Among the problems these affordances might help solve are satisfying needs related to warmth, protection, food, and sex. In order to work out our idea further, we will now address some of the simplest pulls (not focusing on more complex problems like cultural coordination) onto the person from the social environment, that is, the provision of resources that help solve basic problems in a variety of manners (like providing body heat, breast milk, protection from predation, and making tradeoffs in energy conservation).

## Answering the Organism’s Metabolic Needs: Core Functions of Homeostasis

Many of the problems that attachment solves are related to energetic balance, and the organism can accomplish energetic balance through homeostatic regulation. Thus, direct proximity maintenance is critical to maintaining and/or reinstating the affordances of the social unit, allowing each individual animal more flexibility and capacity to meet their own homeostatic needs. Nelson and Panksepp (1998) suggest an integrated social-emotion system based on the neural circuitry supporting distress vocalizations mediating separation distress and a complementary social reward circuit mediating contact comfort. Specifically, they suggest that mechanisms that sub-served thermo-regulation, energy balance, and place-attachment were exapted for use in a social engagement circuit and mechanisms for pain were exapted for use in a separation distress circuit. From this perspective, many of the core components of attachment behavior can be understood as *radically embodied, homeostatic systems.* The perception of affordances thus serves to maintain neutrality in those systems.

Assuming that attachment processes have exapted from more primitive thermoregulatory, harm avoidance (e.g., pain), and energy balance (e.g., nutritive and peripheral arousal control) mechanisms, one can re-envision attachment as an embodied system designed to maintain a balance between proximity to familiar and predictable others, and exploration of the environment. If the individual’s interoceptive, exteroceptive, stress, and thermoregulatory states are at baseline, then exploratory behavior is the norm. If not, then proximity seeking should be the norm. From this perspective, the physiological state of the person plays a central role in determining attachment behavior. If true, then attachment behavior should be modifiable via the manipulation of that physiological state as much as it is by psychological context. Such a hypothesis would be most strongly tested in the context of thermoregulation, peripheral arousal, or hunger given that all of these states can be manipulated to a reasonable extent without an actual threat or crisis. Thus, if physiological changes in a safe and otherwise comfortable environment trigger support seeking and proximity maintaining behavior, then the embodied approach is likely to be fundamental to attachment.

In the next section, we suggest that embodied mechanisms related to homeostasis support attachment behavior and the balance between proximity seeking and exploratory behaviors. To explore this embodiment hypothesis, we first outline how proximity maintenance behaviors can be regulated in an embodied system. Then we discuss what is known about the mechanisms that support the switch from proximity maintenance to exploratory behavior, entertaining the notion that the push and pull dynamic between proximity seeking and exploration can be understood as an embodied process mediated by non-representational cognition. Finally we discuss how individual differences, the aspect of attachment theory most strongly related to representational cognition, might emerge not from representational processes, but through individual differences in physiology, and changes in embodied systems through epigenetics and conditioned learning.

## Regulating the Organism’s Social Baseline Efficiently through Homeostasis: The Architecture of Radical Embodiment

In many instances the non-human animal-based neurobiological literature can sketch the grounds for a radically embodied framework in humans. Mental representations are rarely used as mediating mechanisms, and most of the animal literature instead focuses on how external information, such as touch or caregiver distance, promotes specific behavioral responses from the animal. Moreover, the mediating mechanisms from perception to action are frequently described at a physiological level, fully constraining the problem without reference to computation or representation. Here we seek to provide a more detailed understanding of the underlying neurobiology, and point to how representational mechanisms are not (yet) needed to understand basic attachment mechanisms, ranging from proximity maintenance (for safety and thermoregulation) to exploration, a quintessential attachment behavior. From there we move on to potentially higher order mechanisms that could integrate radically embodied mechanisms with predictive control.

### Proximity Maintenance and Maintaining Security

Altricial animals have developed many capacities to signal their needs related to homeostatic and survival problems. Crying is a key and useful behavior in studying attachment dynamics (Bowlby, 1969/1982; Gracanin et al., 2014). In animal research distress vocalizations are frequently used as a model of crying (Panksepp, 1998) and have led tractability to questions difficult to answer in humans. The majority of these processes can be described without the use of internal representations and thus suggest many attachment processes don’t require internal representations to function.

Most highly social animals go through a multi-phase response to maternal separation (Bowlby, 1969/1982; Mineka and Suomi, 1978). These include a protest phase involving distress vocalizations, and a despair-phase marked by quiet and depressed behavior. Both short-term and long-term separation responses have been heavily studied in animal models using distress vocalizations. Short-term social isolation in rats is associated with increased activity, heart rate, respiration, corticosterone release (a stress substrate), and ultrasonic vocalizations (Shapiro and Salas, 1970; Hofer, 1984; Stanton et al., 1987). Long-term isolation is associated with decreases in growth enzymes, heart rate, and behavior and increases in stress response (Hofer, 1987; Kuhn et al., 1990; Rosenfeld et al., 1992).

Hofer’s (1987) research suggests that these specific effects of isolation can be modified or eliminated by the introduction of sensory stimulation mimicking the softness, warmth, and other sensory features of the dam and littermates (Blumberg et al., 1992). Thus, sensory information appears to be sufficient to constrain the animal’s behavior in relation to its homeostatic needs, and no representation appears to be necessary. Moreover, Harvey and Hennessy (1995) have found an inverse U-shaped relation between corticotrophin releasing hormone and distress vocalizations indicating that low and high levels of stress diminish distress vocalizations. Such a finding is in line with what one might predict from an ecological perspective. Vocalizations and activity should presumably be useful if a caregiver is proximal enough that proximity maintaining behaviors will elicit help in time, however, as stress builds, it is more dangerous to continue vocalization and activity with no response due to the risks of predation. Thus, high levels of stress may serve to provide information that the caregiver is not in a position to help, and therefore radio silence is preferred.

Hofer’s (2006) research has been instrumental in determining how sensory stimulation socially regulates homeostatic needs in laboratory animals. Hofer (2006) notes that attachment better refers to a number of basic processes that tie sensory stimulation directly to physiological regulation and behavior in a manner that promotes the formation, regulation, and maintenance of sustained social relationships. His lab has identified a critical mechanism by which odor preferences can be conditioned in rat pups (Sullivan et al., 1986). Stroking a pup with an artists brush for 5–15 min while simultaneously presenting it with a neutral odor leads to clear preferences for the odor during test. This procedure has been suggested to parallel maternal odor and maternal grooming behavior.

There are a number of direct behaviors that relate to homeostatic needs, and neurobiological markers that motivate the organism. For example, the sensory pattern induced by maternal grooming behavior is associated with endogenous opioid release (Panksepp, 1998, also see the discussion below) in most mammals. Endogenous opioids are critical substrates for consummatory reward (Levine et al., 1985), which is associated with bringing the organism back to homeostasis related to primary drives, such as those governing sex, hunger, and thirst. Furthermore, ultrasonic vocalizations are also inhibited by opioid agonists in a variety of species (Herman and Panksepp, 1978; Kalin et al., 1988; Carden et al., 1991, 1994) with μ-opioid receptors the likely mediators of this effect. Supporting the specificity of these effects, opioid antagonists potentiate distress vocalizations (Herman and Panksepp, 1978, 1981; Panksepp et al., 1980b, 1985; Kalin et al., 1988), and milk creates the analgesic and behavioral effects associated with opiate administration (Smotherman and Robinson, 1987; Blass and Fitzgerald, 1988).

One can imagine that sensory information such as odor and touch get integrated. From there, reward gets associated with odor through opioid or other reinforcement mechanisms, and behavioral approach motor outputs are associated with odor in the brain. The odor stimulus thus becomes directly paired with an approach motor output, making the odor an attractor for the animal. As this example indicates, much of this process requires no mediating representation to understand the regulation of homeostasis. Further, this description has the added benefit of greater specificity regarding the process, and allows for greater understanding and control of attachment behaviors than would traditional representational approaches.

In humans, Coan et al. (2006, 2013) and Johnson et al. (2013) have found that handholding diminishes the neural response to threat. In these studies a reliable network of brain regions are activated to the threat of shock. Generally, activity in this network is reduced in the partner handholding condition (sometimes the partners are friends, other times romantic partners), and the extent to which there are reductions in activity is related to the quality of the relationship. Cognitive neuroscience models typically predict that such effects will be the result of neural regions involved in emotion regulation, facilitated by internal working models, becoming more active and down-regulating the threat matrix. Yet, numerous studies (Coan et al., 2006, 2013; Johnson et al., 2013) have found no increased activation in self-regulatory regions, nor any other brain region. This evidence is consistent with the hypothesis that it is not that social contact necessarily diminishes the threat response *per se*, but rather scales perception of the threat so that the threat response is muted. While this can be mediated through physiological changes, it is also hypothesized that this muting is also a function of perceptual scaling of the magnitude of the threat.

One of the prime indicators that proximity is at a neutral, and desired, level is body temperature. This is probably because lack of appropriate thermoregulation in people means certain death (Blatteis, 2001). In human infants this is particularly critical as they lack the surface to body ratio to self-regulate their temperature in the same manner as adults. Thus, human parents provide the infant with an essential source of thermoregulation. In fact, skin-to-skin contact (as compared to swaddling in a cloth) leads to less skin temperature reduction just after being born (Bystrova et al., 2003). Specifically, adults help infants stay in a “thermoneutral zone” (i.e., to maintain homeostasis; Cannon, 1929), while simultaneously reducing the net energetic expense of warming the body. Further, the secretion of oxytocin, a precursor to the process that leads to vasodilation and increases in skin temperature, helps in reducing the total energy expense of two individuals by activating the endocrine system (Eriksson et al., 1994). Thus, social contact maintains a kind of temperature homeostasis in the most bioenergetically efficient manner (see also IJzerman et al., 2015b).

Examples of social thermoregulation exist in the animal and human literatures, and, again, there are physiological correlates that should be able to explain many classical attachment effects. For example, individual differences may point to mediating representations, but there may be physiological differences between individuals that could also explain such effects. That is, hairless mice produce less brown adipose tissue (BAT) during cold snaps if they aggregate with other individuals. Producing less BAT helps them manage temperature and leads to savings in bioenergetics by decreasing the need for fat stores, and, in turn, the development of one’s body may contribute to social interactions later in life. Similarly, marmots have a significantly higher chance of survival if huddling (crucial to social thermoregulation) occurs with the co-presence of lower ranking marmots in the group (Armitage, 1999).

Notably, all of these examples describe direct processes for proximity maintenance (in service of homeostasis), eliminating the need for internal representations. The proximity of conspecifics is critical for an animal’s assessment of its energetic balance, and its ability to meet homeostatic needs and thus scales perception of the environment in proportion to those resources. As such, social proximity is a signal that the animal may switch from defensive processes and focus on affiliation and/or exploration. But how does that switch occur, and can the switch flip via embodied processes?

### Moving to Exploration and Energetics

Porges (2007) argues for a distinction between two segments of the vagal nerve, one evolutionarily newer, and one older. The phylogenetically newer vagus serves as a brake by which vagal output to the heart allows the animal to switch between a mobilized state and a calm state (Porges, 2007). When vagal tone is high, the vagus inhibits heart rate, but low vagal tone releases this break resulting in disinhibition. Thus, the vagal system is central to the peripheral control of the action state of mammals. This adaptation allows an animal to engage in behaviors that would be dangerous with a strictly reptilian vagus. Organisms have three types of reactive defense behaviors that significantly and rapidly alter autonomic nervous system function, fight, flight, and freezing (Porges, 2011). Fight and flight both involve an increase in sympathetic output, mobilizing resources for vigorous muscle movement and physical action. Freezing, alternatively, involves immobilization with fear. This state can be accompanied with dramatic and dangerous drops in heart rate and blood pressure. Social engagement (e.g., sex, breastfeeding, childbirth) requires a level of immobilization without fear, or staying calm in the presence of and in interactions with others. Porges (2007, 2011) believes the mammalian vagus to have evolved just for such functions.

Moving onto how the organism engages in exploratory behavior, it helps understanding the role of the hormone oxytocine (OT). OT may be critical in regulating which part of the vagus is active, and the downstream functions of the vagus. Oxytocin is a peptide hormone that functions as a neuromodulator in the CNS (Carter, 2014). OT is released during social engagement of various kinds (Wakerley and Lincoln, 1973; Keverne et al., 1983; Uvnas-Moberg et al., 1993) including nursing, vibrotactile, and thermal stimulation. Notably, OT has an excitatory effect on social motivation as it is associated with the onset of maternal behavior, increased sexual response, grooming behavior, and physical contact (Pedersen and Prange, 1979; Argiolas et al., 1987; Gorzalka and Lester, 1987; Witt et al., 1990; Witt and Insel, 1991; Winslow and Insel, 1993; Pedersen et al., 1994).

Furthermore, OT is now well documented to be a critical mediating mechanism in vole pair bonding (Cho et al., 1999; Bales et al., 2004). It mediates maternal behavior (Bosch and Neuman, 2012), protects against negative consequences of social isolation (Grippo et al., 2009), and is critical in both birth and lactation (Carter, 1998). In humans, oxytocin has been related to many social behaviors as in other mammals, but also more “abstract” sociality such as that found in economic trust games (Kosfeld et al., 2005; Zak et al., 2007). Recent studies have begun to reveal a direct link between OT and the other previously mentioned homeostatic processes, namely thermoregulation and metabolism (Kasahara et al., 2007, 2013; Takayanagi et al., 2008; Chaves et al., 2013). Chaves et al. (2013) found support that OT has inhibitory effects on carbohydrate preferences and is associated with rectal temperatures. In line with these findings Katsuhiko Nishimori and colleagues note that OT receptor knockout mice have higher rates of obesity later in life (Takayanagi et al., 2008) and impaired thermoregulation capacities (Kasahara et al., 2007). Moreover, those deficits can be eliminated with the introduction of OT receptors in thermoregulatory regions of the hypothalamus. This research suggests that social contact might have important downstream implications regarding the expenditure of energy on exploratory activities. Indeed, we suggest that OT and the vagus are important in this process, acting as a bridge between defensive states and calm states, and are key mediators of the switch from defensive behavior, proximity seeking behavior, and exploratory behavior.

This review suggests that the basic mechanisms involved in attachment behavior can be understood without mediating representations. At its core, attachment is about harm avoidance, thermoregulation, and the switch from maintaining security toward exploratory states. The more we learn about the neurobiological mechanisms that control such behavior, the clearer it becomes that mediating representations are unnecessary to explain this behavior. As such, the normative processes that support attachment do not seem to require a representational architecture, but what about individual differences? The biggest challenge for a radical embodiment account of attachment is the development of individual difference. Individual differences in attachment, more than any other element, are understood explicitly as representational in nature. Next we discuss how embodied processes can also explain these aspects of attachment relationships.

### Embodiment of Individual Differences in Attachment

Normative attachment processes can be understood quite well from an embodied perspective. Yet, it is within the study of attachment styles that representations come to the fore. A simple search for “attachment representation” in any scholarly search tool will produce hundreds, if not 1000, of papers with a central theme of the representation of individual differences in attachment. For example, Sroufe (2005, p. 365) notes that “Not only does representation mediate the effect of prior experience on later adaptation, later experience and adaptation impact representation.” The one particularly important issue to address before accepting attachment as radically embodied is individual differences.

Thus far, we have argued that many processes in attachment are primarily homeostatic in nature. Many homeostatic mechanisms, like those that control eating and sex, are not based on a fixed set-point, but rather on the positive-incentive value of the behavior (see Berridge, 2004). From this perspective, any of the physiological systems in which attachment behavior helps regulate will act as a signal to gain social contact. Given that no set-point assumption is necessary, the fact that individuals vary on how much they seek social contact, and the relative reward value of that contact for the individual is not surprising. Many of these earlier effects may well be explained through systematic differences in the organism’s physiology.

That is, physiological and sensory cues of safety versus environmental challenge may underlie the triggers for attachment behavior. Thus, the baseline activity, strength, and potency of underlying neural substrates might be the first and most important way in which individual differences in attachment behavior emerge. For example, opioid tone might influence overall attachment and social behavior (Panksepp, 1998). Low opioid tone may be associated with excessive social support seeking, just as the introduction of opioid antagonists produces social behavior in social animals (Herman and Panksepp, 1978; Panksepp et al., 1980a; Keverne et al., 1989). Moreover, low opioid tone has been demonstrated in individuals with borderline personality disorder (Ripoll et al., 2013), and activity in the A118G polymorphism of the mu-opioid receptor gene has been associated with avoidant attachment, affectionate relationships, and sensitivity to social reward (Troisi et al., 2010).

Differences in oxytocin function may also be critical in determining attachment behavior. For example, oxytocin receptor gene (OXTR) variation is associated with prosocial behavior (Israel et al., 2009), empathy and stress reactivity (Rodrigues et al., 2009), pair bonding (Walum et al., 2011), parenting behavior (Bakermans-Kranenburg and van Ijzendoorn, 2011), emotional loneliness (Lucht et al., 2009), and functional links between hypothalamic and limbic system activity in emotional face processing (Tost et al., 2010). Epigenetic variation in methylation of the OXTR gene is similarly associated with neural responses to ambiguous social stimuli (Jack et al., 2012), autism and callous-unemotional traits (Gregory et al., 2009; Kumsta et al., 2013). Similarly, the work by McGowan et al. (2009) indicates that methylation of the hippocampal glucocorticoid receptor gene is influenced by social factors and influences exploratory behavior and stress responses well into adulthood.

Although the individual difference component of attachment is the part of attachment theory and relationship theories more broadly that are most consistent with representational mediating processes, this discussion points to several possible avenues through which representation is unnecessary for the emergence of individual differences. From this perspective homeostatic mechanisms based on positive-incentive value (and therefore related conditioning processes as well; see Beckes et al., 2010, 2013; Beckes and Coan, 2014) can explain a great deal of variation in attachment behavior. Moreover, the patterns of early attachment relationships laying a foundation for later development are clearly identifiable with this approach through the development of physiological differences, epigenetic processes, and conditioned learning.

How does this work for exploration beyond the direct social environment, and for prediction of the future? Many attachment behaviors should be about predicting future events as much as reacting to current events. In addition, the switch from maintaining security to exploratory behaviors has important downstream implications regarding the expenditure of energy on exploratory activities, and SBT (Beckes and Coan, 2011) suggests that differences in attachment are important in budgeting energetic resources for exploration, as energy is highly sensitive to social resources. Indeed, humans have a great capacity for prospective cognition, often thinking about future events, possible outcomes, the perspectives of others, and removing themselves cognitively from the immediate environment. If cognition is solely driven by the interaction between the organism and its environment, how does such internally driven cognition emerge?

## Individual Differences: The Need for Prospection

The biggest challenge for radical embodiment theories is to move from exploration to prediction. How do temperature predictions, for example, relate to skin temperature changes? Are such notions predicated upon the idea of representing abstract concepts, as postulated by Lakoff and Johnson (1999)? One hallmark study by Boroditsky and Ramscar (2002) found that time can be grounded in the concrete experience of space. However, Srinivasan and Carey (2010) found that comparable time-space effects are already detected in preverbal infants. Thus, such abstract representations might be explainable through either embodied processes, or a process of scaffolding (see also IJzerman and Koole, 2011; IJzerman et al., 2015b).

Scaffolding theories suggest that associations between social experiences and bodily conditions create the groundwork for later models of the world (Piaget and Inhelder, 1969; Mandler, 2004; Williams et al., 2009). Physical contact between two bodies creates a number of bodily states that serve as the basis for grounded relationship metaphors. Contact through sex, breastfeeding, hugging, handholding, and intimate contact all produce sensory stimulation of mechanoreceptors and thermoreceptors in the skin. Indeed, the link between softness and warmth on the one hand, and basic social contact on the other may be both genetically prepared and heavily reinforced early on in life, and re-presented in attachment styles later in life (Caporael, 1997; Damasio, 1999; Fiske, 2004; Cohen and Leung, 2009; IJzerman and Semin, 2010; IJzerman and Cohen, 2011). This presents at least two important questions. How much of attachment is radically embodied and how much is representational? And how might scaffolding occur?

To date it is unclear how scaffolding could occur. One potential candidate is the theory of PARCSs (Tops and Boksem, 2011; Tops et al., 2014), which suggests two levels of control, one predictive and one reactive. According to PARCS, radical embodiment may be the rule for reactive control systems, however, predictive control systems are re-presentational in nature. From this perspective motivational control can be shifted between predictive systems and reactive systems, which allows for representational influence on behavior, but that representational structure is fundamentally connected with an embodied reactive control system. In this way the representational architecture of the mind (a soft representation; Zajonc and Markus, 1984) is built in close association with the embodied architecture, which leads to this scaffolding phenomenon. A touch from another person may thus contribute not only to the feeling that the world is secure, but that the environment also has sufficient resources. From that perspective, the organism can make predictions about what future action to take. This approach thus allows for clear predictions about when representational processes are being used, and the neural mechanisms that support them. In order to elucidate this idea, we now explore how radically embodied cognitive systems interact with representationally based cognitive systems, and how representational systems may have evolved to support relationship cognition.

### Integration of Radical Embodiment and Representation: Theory of Predictive and Reactive Control Systems

One fascinating finding is that maternal thermoregulation in rats may lead to greater brain growth. And, we know that secure attachment styles are related to greater self-complexity in humans (e.g., Mikulincer, 1995). Could it be that on top of the proposed radically embodied architecture, there are predictive models for the benefit of greater exploration? This idea of stable individual difference patterns of attachment behavior has always been one of the hallmark features of attachment theory. Notably, Bowlby (1969/1982) theorized that these differences – and their stability throughout life – were due to *internal working models*, or representations of the relationship with the caregiver.

One possibility is that the brain has evolved additional systems for representational cognition that rely on radically embodied architectures in the brain, in part because of an increase in sociality over evolutionary time. Notably, brain size and the size of social groups across mammals, particularly primates, are positively correlated (Barrett et al., 2002) in such a way as to indicate a link between neocortical volume and sociality that is meaningful. Dunbar and Shultz (2007) argue that this relationship was promoted because increased social group size and monogamous pair-bonding required greater predictive capacity to navigate social relationships. Indeed, it may be that in evolutionary history greater sociality led to pressures that promoted increased cortical growth while simultaneously increasing energetic efficiency through social cooperation.

Why might this be the case? We think that the human brain evolved greater prospective/predictive capacities in order to promote planning and simulation of possible outcomes due to an increase in the need to predict the behavior of others that emerged with increased sociality, like more complex “meta-relationships” (Fiske, 2012; Bohl, 2014). In order to predict the future of relationships and social partnerships, greater prospective capacity became essential so that one could make appropriate tradeoffs between current outcomes at a selfish level and the potentially greater reward from working cooperatively with others, more or less like a “weather report” of the social environment (IJzerman et al., 2015b). This tradeoff and balance requires the ability to determine the trustworthiness of others and predict their behavior as a function of varied situations. This happens in two ways. First, predictive cognition was promoted and, as a function, brain size likely grew. Moreover, increased cooperative behavior also promoted energetic efficiency – a positive weather report – allowing for greater exploration of the environment, promoting multiple levels of switches, controlled in part via OT mechanisms, between exploratory and defensive behavior, and predictive (representational) and reactive (embodied) cognition.

In line with this reasoning, PARCS suggests that reactive control systems evolved early in evolutionary history for the purpose of behavioral control in unpredictable environments. This system is composed of lateral limbic system structures such as the ventral striatum (VS), anterior hippocampal formation, and amygdala, as well as ventrolateral cortical structures such as the inferior frontal gyrus (IFG), and anterior insula (AI). This system is thought to specialize in the processing of novelty, (cf., Whalen, 2007), biological salience (cf., Adolphs, 2010), and urgent environmental stimuli in order to react to exigencies. It functions in a feedback-guided manner to the immediate situation and focuses attention narrowly on the local situation. Thus, when the organism is lonely, it will likely seek for warm and protective others. In this manner the organism can take new information and communicate with predictive systems to update internal predictive models promoting greater predictive control in the future (Hasher and Zacks, 1979; Tops and Boksem, 2011).

Predictive control systems, on the other hand, are comprised of dorsomedial structures such as the posterior cingulate cortex (PCC), precuneous, angular gyrus, parahippocampal cortex, posterior hippocampal formation, medial prefrontal cortex, and dorsolateral prefrontal cortex. It is believed that this network of systems is largely an outgrowth of evolutionary pressures that emerged in highly predictable and stable environments (Tops et al., 2014). This system supports a variety of cognitive functions that might be representational in nature, and is highly intertwined with the reactive system. PARCS theorizes that the system is largely composed of neural structures intrinsic to the default mode network (DMN), such as the posterior cingulate, precuneous, medial temporal lobe, and medial prefrontal cortex (Raichle et al., 2001) as well as dorsal executive regions. It is involved in cognitive tasks with internally focused attention such as imagining a different time or space (Buckner and Carroll, 2007), or another person’s perspective (Waytz and Mitchell, 2011). Craik (1943) suggested that imagining the future using internal models allows for testing alternative possibilities, and making better predictions regarding situational outcomes. In the same sense, PARCS suggests that the dorsal predictive system function is to run simulations to predict future events, and, in line with the rest of our paper, likely to “gage” the amount of resources available (see also IJzerman et al., 2015a). Thus, the dorsal predictive system engages in creating internal models that predict future outcomes through simulation, and updates those models slowly, in line with the idea that it responds to environmental predictability.

A key idea is that the predictive control system creates simulations largely based on information from embodied sources. Thus, much of the brain may function in a radically embodied manner, but predictive control has required a form of representational architecture that uses simulation processes, and engages in computational processes. Soft representations are scaffolded upon embodied architecture, but still, nonetheless, may exist, and be the basis of internal working models. In order to test such a hypothesis, falsifiable predictions must be produced that can be used to challenge this model.

## Solving the Puzzle

In order to determine which way of thinking about attachment is most accurate, one must develop alternative hypotheses that can be derived from each approach. Some possible ways of looking at extant data could provide compelling evidence for one alternative or the other with supplementation of a few critical experiments. Another possibility is that we have not developed enough knowledge of the underlying systems to move to a fully radically embodied approach. As Chemero (2009) notes, mediating representations are often used as placeholders until a system is understood with enough fidelity that one no longer needs those placeholders. But what does the current evidence indicate?

### Current Evidence and Competing Alternatives

The key to distinguishing which alternative is more accurate, the PARCS model or the radically embodied model, may lie in the exigencies of the situation. If soft representations are scaffolded onto embodied processes, but are primarily for adapting to predictable circumstances, then the predictability of the circumstance is likely the key distinguishing variable between the control of internal working models and radically embodied processes. From this, one can assume that reactive control will become dominant in distressing circumstances, whereas predictive control should be dominant in non- or eustressful circumstances (Selye, 1974). From there, specific hypotheses can be devised to challenge each approach to determine if the empirical facts fit the theoretical model.

Current research regarding internal working models provides clear support for a reactive system, which does not require internal representational structures. For example, conditioned learning procedures have been used to create attachment associations in implicit paradigms that appear to require no underlying representational mediation, and are instead built on basic associational and reinforcement processes (e.g., Beckes et al., 2010, 2013). Moreover, the idea that predictive systems (internal working models) are critical in acute attachment behavior is questionable given that differential attachment behavior tends to emerge during situations of distress, when the reactive system should be dominant (Simpson and Rholes, 2012). For example, Simpson et al. (1992) found that behavioral differences between avoidant and secure women did not emerge without fear or anxiety. Similarly, Simpson et al. (1996) found that the quality of a conflict discussion was related to attachment anxiety only in circumstances in which couples were discussing a major problem, and thus experiencing acute distress. These findings are core to Simpson and Rholes (2012) diathesis stress model of attachment, suggesting that attachment styles really emerge in distressing circumstances, with few observable differences between anxious, avoidant, and secure people in low stress circumstances.

This provides intriguing support for the PARCS approach. On the one hand, internal working models appear to emerge in conditions in which one would expect the reactive control system to be the primary driver of behavior. This would suggest that attachment styles are largely reactive, and therefore embodied. Alternatively, it also indicates that in low stress circumstances a calmer, potentially predictive system, may be guiding behavior. This indicates that the predictive system exists, and that internal representational processes may be involved in attachment, but that they do not matter much in terms of mediating behavior in circumstances that are acutely distressing.

In this context, it is important to note that stress is not equivalent with unpredictability. Early experiences may determine whether challenges or environments are perceived as predictable. Or, whether there are internal models available to meet the challenge. Insecurely attached individuals will tend to revert to reactive control when challenged, while securely attached individuals may sustain predictive control (and predictive homeostasis) enabling them to activate internal working models. This dissociation between stress and predictability may be shown in oxytocin function: OT is usually associated with anxiolytic effects, but intranasal OT increased anxiety in response to an unpredictable stressor (Grillon et al., 2013).

One possibility consistent with PARCS is that attachment security may require the action of the predictive system, having emerged out of predictable attachment relationships, whereas insecure attachment is largely controlled by the reactive system. Such a conceptualization would fit with the extant literature regarding stress-diathesis (Simpson and Rholes, 2012). From this perspective, distress induced attachment behavior starts from reactive control, but shifts to predictive control in the case of secure attachment (Tops et al., 2014). There are a few strong hypotheses one can make if the PARCS prediction is correct. Each type of prediction can be organized by methodological approaches, each of which can be used to test critical predictions from the PARCS model. First, the hypothesis that predictive control systems update slowly relative to reactive systems, and that secure attachment is predictive whereas insecure is reactive, provides an opportunity to test the PARCs model using learning methods, and in particular conditioning paradigms. Second, the hypothesis that secure individuals should perceive challenges as more predictable, and shift to predictive control relatively quickly can be tested using neuroimaging and psychophysiological methods looking at the relative activity and time course response of lateralized brain activity, medial prefrontal regions, AI, and posterior insula. Third, the prediction that the reactive system is radically embodied can be used to predict enhanced effects during an unpredictable stressor in an embodiment paradigm.

### Conditioning Paradigms

If the PARCs prediction that secure attachment is prospectively controlled and representational, whereas insecure attachment is reactively controlled and non-representational is correct, then secure and insecure attachment associations should be variably modifiable using conditioning procedures. As PARCS notes, predictive models are updated more slowly, and are thus more resistant to change relative to reactive models. If true, secure representations and associations should be harder to eliminate than insecure representations. Laboratory methods have been used to manipulate attachment styles using negative reinforcement (e.g., Beckes et al., 2010, 2013). In these studies a threatening stimulus (e.g., images of snakes, shock threats) is paired with the images of another individual in a manner that produces negative reinforcement as measured by attentional biases and lexical decision measures using faces as primes for attachment relevant words. Presentation of the faces occurs after the offset of the negative stimulus, or presented after an operant procedure in which the person asks for help (from the person in the photo) while under threat of shock, pain, or another negative stimulus. Consistent, warm responses (continuous reinforcement) in such procedures produces secure associations with novel faces. If, however, the other is inconsistently responsive, producing a variable ratio reinforcement schedule, such a procedure should produce anxious/ambivalent attachment associations and hyper support seeking behavior. It could be possible to use such a procedure repeatedly to produce such attachment associations and then test the ease with which one can shift those associations in later trials, predicting that insecurity is easier to shift than security.

Alternatively, and more externally valid, one could use such techniques to see if people’s attachment styles in a specific relationship are modifiable through such procedures, and compare the relative ease of modifying such styles in secure vs. insecure individuals. Moreover, one might be able to use the basic principles in more realistic situations, further improving external validity, such as producing situations in which each person is reliant on the other to avoid some undesirable outcome. In addition, researchers could use such principles to alter attachment dynamics in intact relationships. For example, emotionally focused therapy (e.g., Johnson et al., 2013) has at its core a process of training partners in distressed relationships how to respond consistently to their partner’s needs. Research into this method of intervention indicates that such an approach produces decreases in attachment insecurity, and improvements in social emotion regulation over a reasonable period of time. Additionally, one might be able to use physical warmth cues as an unconditioned reinforcer in some situations. Raison et al. (2015) propose full body warmth as a treatment modality for affective disorders. It could also be an effective method of promoting security in attachment relationships if the partner is paired with hyperthermia consistently over a period of time. Finally, one could track intact relationships over time measuring responsiveness during stressful situations, attachment styles, and changes in those dynamics to see if changes in responsiveness predict more change in insecure individual’s attachment styles relative to secure individuals.

### Neuroimaging and Psychophysiological Methods

Using PARCs, one can predict specific patterns of response from neurobiological measures. As noted above, challenges should lead to predictive control more easily for secure individuals than for insecure individuals. Moreover, when confronted with a challenge, secure individuals should react with reactive systems, but rapidly switch to predictive control using predictive models to cope with the challenges. For example, when faced with an acute stressor secure individuals should show a shift from right hemisphere control to left hemisphere control, and then to dorsomedial control over time (Tops et al., 2014). This is consistent with going from right hemisphere reactive emergency control (orienting, vigilance, etc.), to left hemisphere reactive control in coping (e.g., reappraisal), to predictive control (medial mechanisms). One might predict, for example, a more intense AI activation and slower switch to medial prefrontal activation in insecure individuals relative to secure individuals, indicating less contextualized perception of threat in those individuals. These questions could be explored regressing attachment security on BOLD activity of AI and medial prefrontal regions in an fMRI threat paradigm. Such a prediction should be most evident when an individual is faced with a social or relationship threat. Several different types of paradigms could be employed to test such a hypothesis. For example, using EEG one might find that relationship threats produce relatively greater left frontal alpha power shortly after the threat, but over time that shifts to relatively greater right frontal alpha power in secure individuals more quickly than in insecure individuals (see Coan and Allen, 2003).

Notably, the effects of embodiment manipulations through reactive and predictive systems may further depend on the required level of differentiation and contextualization of interoceptive information. Reactive control in emergency situations is associated with an integrated perception of momentary resources and arousal in the AI, at the cost of differentiated perception in the posterior insula. By relating the differentiated viscerosensory representations in the posterior insula to the predictive control system, PARCS suggests that activation of these areas relates to increased interoceptive self-observation skills and fine-tuning of perception and internal state. For instance, a recent study found that the response of the taste-sensitive region of the posterior insula to food images was directly related to the body’s homeostatic state as indexed by levels of peripheral glucose (Simmons et al., 2013). By contrast, PARCS predicts that urgency, emergency, and unpredictability activate reactive control and the AI and suppress predictive control. For instance acute stress and reactive control inhibit and subsequently increase food intake (Bazhan and Zelena, 2013), while predictive homeostatic control matches hunger feeling to energetic state and circadian rhythm (Moore-Ede, 1986; Simmons et al., 2013). The association of reactive control with anxiety explains why anxiety is associated with increased undifferentiated awareness of arousal (physiological activation; Pollatos et al., 2007) but less differentiated awareness of specific somatic states (somatic neglect; Koole et al., 2014). It also explains the negative correlations of trait internal state awareness with social anxiety, depression, psychological distress, and external control, and more accurate and extensive self-knowledge (Watson et al., 1996; Trapnell and Campbell, 1999; Ghorbani et al., 2004; Takano and Tanno, 2009). This provides a way in which neuroimaging methods, by investigating insular involvement in embodied processes (posterior vs. anterior), might be used in combination with embodiment approaches to further test the PARCs model.

### Embodiment Paradigms

Finally, one might predict that embodied manipulations will have stronger influences on behavioral responses when unpredictable stress is higher, particularly in insecure individuals. The manipulation of the stress and predictability of the situation, with the embodiment of a representation might offer some compelling examples of how and whether predictive systems interact with embodied systems. For example, the embodiment effects of a warm cup of coffee increasing the perceived warmth of another person (see Williams and Bargh, 2008) may be stronger if manipulated during a period of unpredictable shock threat or after a brief and unpredictable stressor.

As these example indicate, several types of hypotheses can be generated to differentiate a PARCs approach to a radically embodied approach. Although the jury is still out on which theoretical approach is better at capturing the data, such predictions give us a set of tractable questions to begin testing this question.

## Conclusion

**FIGURE 1:**
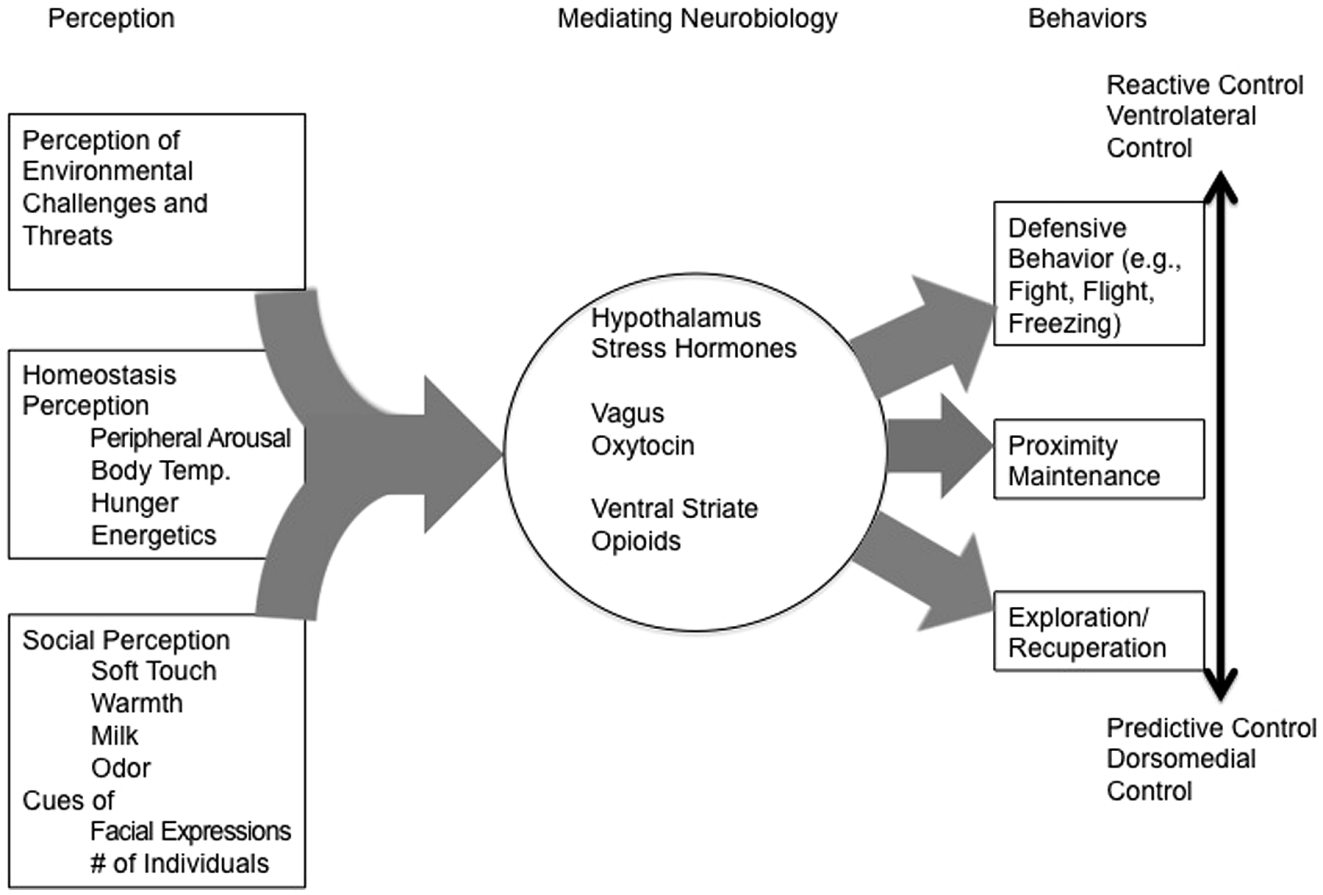
**It provides a simplified version of the proposed neural mechanisms of attachment, from input (perception) to processing (mediating neural mechanisms) to output (behavior) of the entire spectrum of what we understand as “attachment.”** The figure is necessarily simplified for the sake of supporting our written description. For example, bidirectional flow is assumed, but not depicted. Input related to homeostatic imbalance promotes stress system activity with frequent release of oxytocin promoting its output (e.g., defensive behaviors), and this relationship is mediated by relevant neurobiological mechanisms central to reactive control (ventrolateral structures). Input related to homeostatic balance push output (e.g., exploration or reparative behaviors) toward predictive control composed of primarily of dorsomedial structures.

Herein we have described a number of psychological and neurobiological processes related to attachment from a radically embodied perspective (see **Figure 1**). Many aspects of attachment are well suited to be described and understood from a non-representational perspective and should be much better explored than they currently have been – preferably through dynamical models. Specific sensory systems have been linked to many of the physiological effects of social contact, with different sensory and nutritive stimuli regulating peripheral physiology in predictable manners. Further, neural systems involved in responding to social isolation and separation are similar in structure to physical pain circuits, regulated by the same opioid mechanisms. Also, mammals appear to have social specific functions related to the regulation of visceral organs and communication social communication processes through the vagus nerve. This parasympathetic circuit directly ties perception of social targets to the regulation of metabolic, stress, and emotion circuits. Moreover, this system appears to have OT controls located in the amygdala, hypothalamus, and directly in the brainstem nuclei that the vagus originates in. OT itself is intrinsically linked with the control of social behavior, thermoregulation, and metabolic processes indicating even more significant overlap in the neural bases of these systems. We suggest a theoretical approach to thinking about the nature and function of human sociality rooted in conservation of metabolic resources and diminishing the need for environmental defense behaviors. This approach takes a Gibsonian view that social relationships provide affordances to act, or not in some cases, and that removal of those affordances from the environment makes action more challenging, scary, and threatening. Finally, we argue that individual differences in attachment styles need not be conceptualized as representational constructs, but can be thought of as response tendencies that emerge out the person’s unique biological makeup and their learning history.

Disentangling the degree to which processes are representational or computational or not is a simple task. New models that propose a combination of such approaches such as PARCS (Tops and Boksem, 2011) offer a compelling alternative to the all or none approach often represented in the current debates over embodiment. We have endeavored to offer a view of attachment that promotes thinking about the underlying cognitive and neurobiological processes in novel ways, and expands the toolkit we use to approach questions surrounding attachment. The individual and competing hypotheses presented herein should act as a guide to further spark innovation and exploration in this important field of study. In the end all we can do is pursue the question, to what degree is attachment radically embodied?

## Conflict of Interest Statement

The authors declare that the research was conducted in the absence of any commercial or financial relationships that could be construed as a potential conflict of interest.

## References

[B1] Adolphs R. (2010). What does the amygdala contribute to social cognition? *Ann. N. Y. Acad. Sci*. 1191 42–42. 10.1111/j.1749-6632.2010.05445.x 20392275 PMC2871162

[B2] Agnew C. R. Van Lange P. A. M. Rusbult C. E. Langston C. A. (1998). Cognitive interdependence: commitment and the mental representation of close relationships. *J. Pers. Soc. Psychol.* 74 939–954. 10.1037/0022-3514.74.4.939

[B3] Andersen A. M. Chen S. (2002). The relational self: an interpersonal social-cognitive theory. *Psychol. Rev.* 109 619–645. 10.1037/0033-295X.109.4.619 12374322

[B4] Argiolas A. Melis M. R. Mauri A. Gesa G. L. (1987). Paraventricular nucleus lesion prevents yawning and penile erection induced by apomorphine and oxytocin but not ACTH in rats. *Brain Res.* 421 349–352. 10.1016/0006-8993(87)91305-9 2825910

[B5] Armitage K. B. (1999). Evolution of sociality in marmots. *J. Mammol.* 80 1–10. 10.2307/1383202

[B6] Asch S. E. (1958). “The metaphor: a psychological inquiry,” in *Person Perception and Interpersonal Behavior* eds Tagiuri R. Petrullo L. (Redwood, CA: Stanford University Press) 86–94.

[B7] Bakermans-Kranenburg M. J. van Ijzendoorn M. H. (2011). Differential susceptibility to rearing environment depending on dopamine-related genes: new evidence and a meta-analysis. *Dev. Psychopathol.* 23 39–52. 10.1017/S0954579410000635 21262038

[B8] Baldwin M. W. (1992). Relational schemas and the processing of social information. *Psychol. Bull.* 112 461–484. 10.1037/0033-2909.112.3.461

[B9] Baldwin M. W. (1995). Relational schemas and cognition in close relationships. *J. Soc. Pers. Relatsh.* 12 547–552. 10.1177/0265407595124008

[B10] Bales K. L. Kim A. J. Lewis-Reese A. D. Sue Carter C (2004). Both oxytocin and vasopressin may influence alloparental behavior in male prairie voles. *Horm. Behav.* 45 354–361. 10.1016/j.yhbeh.2004.01.004 15109910

[B11] Barrett L. (2011). *Beyond the Brain: How the Body and the Environment Shape Cognition*. Princeton, NJ: Princeton University Press.

[B12] Barrett L. Dunbar R. Lycett J. (2002). *Human Evolutionary Psychology*. Basingstoke: Macmillan/Palgrave and Princeton University Press.

[B13] Barsalou L. W. (1999). Perceptual symbol systems. *Behav. Brain Sci.* 22 577–609. 10.1017/S0140525X99002149 11301525

[B14] Barsalou L. W. (2008). Grounded cognition. *Annu. Rev. Psychol.* 59 617–645. 10.1146/annurev.psych.59.103006.093639. 17705682

[B15] Baumeister R. F. Leary M. R. (1995). The need to belong: desire for interpersonal attachments as a fundamental human motivation. *Psychol. Bull.* 117 497–529. 10.1037/0033-2909.117.3.497 7777651

[B16] Bazhan N. Zelena D. (2013). Food-intake regulation during stress by the hypothalamic-pituitary-adrenal axis. *Brain Res. Bull.* 95 46–53. 10.1016/j.brainresbull.2013.04.002 23590931

[B17] Beals K. P. Peplau L. A. Gable S. L. (2009). Stigma management and well-being: the role of perceived social support, emotional processing, and suppression. *Pers. Soc. Psychol. Bull.* 35 867–879. 10.1177/0146167209334783 19403792

[B18] Beckes L. Coan J. A. (2011). Social baseline theory: the role of social proximity in emotion and economy of action. *Soc. Pers. Psychol. Compass* 5 976–988. 10.1111/j.1751-9004.2011.00400.x

[B19] Beckes L. Coan J. A. (2014). “The distress-relief dynamic in attachment bonds,” in *Bases of Adult Attachment: Linking Brain, Mind, and Behavior* eds Hazan C. Zayas V. (New York, NY: Springer-Verlag). 10.1111/psyp.12056

[B20] Beckes L. Coan J. A. Morris J. P. (2013). Implicit conditioning of faces via the social regulation of emotion: ERP evidence of early attentional biases for security conditioned faces. *Psychophysioly* 50 734–742. 10.1177/0956797610368061 23713682

[B21] Beckes L. Simpson J. A. Erickson A. B. (2010). Of snakes and succor: learning secure attachment associations with novel faces via negative stimulus pairings. *Psychol. Sci.* 21 721–728. 10.1177/0956797610368061 20483852

[B22] Berridge K. C. (2004). Motivation concepts in behavioral neuroscience. *Physiol. Behav.* 81 179–209. 10.1016/j.physbeh.2004.02.004 15159167

[B23] Berscheid E. (2003). “The human’s greatest strength: other humans,” in *A psychology of Human Strengths: Fundamental Questions and Future Directions for A Positive Psychology* eds Aspinwall L. G. Staudinger U. M. (San Francisco, CA: Berrett-Koehler) 37–47. 10.1038/418601b

[B24] Blass E. M. Fitzgerald E. (1988). Milk-induced analgesia and comforting in 10-day-old rats: opioid mediation. *Pharmacol. Biochem. Behav.* 29 9–13. 10.1016/0091-3057(88)90266-3 2832857

[B25] Blatteis C. M. (2001). *Physiology and Pathophysiology of Temperature Regulation*. River Edge, NJ: World Scientific Publishing Company.

[B26] Blumberg M. S. Efimova I. V. Alberts J. R. (1992). Ultrasonic vocalizations by rat pups: the primary importance of ambient temperature and the thermal significance of contact comfort. *Dev. Psychobiol.* 25 229–250. 10.1002/dev.420250402 1624055

[B27] Bohl V. (2014). We read minds to shape relationships. *Philos. Psychol*. 28 674–694. 10.1080/09515089.2014.893607

[B28] Boroditsky L. Ramscar M. (2002). The roles of body and mind in abstract thought. *Psychol. Sci*. 13 185–188. 10.1111/1467-9280.00434 11934006

[B29] Bosch O. J. Neuman I. D. (2012). Both oxytocin and vasopressin are mediators of maternal care and aggression in rodents: from central release to sites of action. *Horm. Behav.* 61 293–303. 10.1016/j.yhbeh.2011.11.002 22100184

[B30] Bowlby J. (1969/1982). *Attachment: Attachment and Loss* Vol. I New York, NY: Basic Books.

[B31] Bowlby J. (1980). *Loss: Sadness and Depression* Vol. 3 New York, NY: Basic Books

[B32] Brewer M. B. Caporael L. R. (1990). Selfish genes vs. selfish people: sociobiology as origin myth. *Motiv. Emot.* 14 237–243. 10.1007/BF00996182

[B33] Buckner R. L. Carroll D. C. (2007). Self-projection and the brain. *Trends Cogn. Sci.* 11 49–57. 10.1016/j.tics.2006.11.004 17188554

[B34] Bystrova K. Matthiesen A. S. Vorontsov I. Widstrom A. M. Ransjo-Arvidson A. B. Uvnas-Moberg K. (2007). Maternal axillar and breast temperature after giving birth: effects of delivery ward practices and relation to infant temperature. *Birth* 34 291–300. 10.1111/j.1523-536X.2007.00187.x 18021144

[B35] Bystrova K. Widstrom A. M. Matthiesen A. S. Ransjo-Arvidson A. B. Welles-Nystrom B. Wassberg C. (2003). Skin-to-skin contact may reduce negative consequences of “the stress of being born”: a study on temperature in newborn infants, subjected to different ward routines in St. Petersburg. *Acta Paediatr.* 92 320–326. 10.1111/j.1651-2227.2003.tb00553.x 12725547

[B36] Cannon W. B. (1929). *Bodily Changes in Pain, Hunger, Fear, and Rage*. New York, NY: Appleton.

[B37] Caporael L. R. (1997). The evolution of truly social cognition: the core configurations model. *Pers. Soc. Psychol. Rev.* 4 276–298. 10.1207/s15327957pspr0104_1 15661664

[B38] Carden S. E. Barr G. A. Davachi L. Hofer M. A. (1994). U50488 increases ultrasonic vocalizations in 3-, 10-, and 18-day old rat pups in isolation and the home cage. *Dev. Psychobiol.* 27 65–83. 10.1002/dev.420270107 8112489

[B39] Carden S. E. Barr G. A. Hofer M. A. (1991). Differential effects of specific opioid receptor agonists on rat up isolation calls. *Behav. Brain Res.* 62 17–22. 10.1016/0165-3806(91)90185-l 1662121

[B40] Carter C. S. (1998). Neuroendocrine perspectives on social attachment and love. *Psychoneuroendocrinology* 23 779–818. 10.1016/S0306-4530(98)00055-9 9924738

[B41] Carter C. S. (2014). Oxytocin pathways and the evolution of human behavior. *Annu. Rev. Psychol.* 65 17–39. 10.1146/annurev-psych-010213-115110 24050183

[B42] Carter C. S. Keverne E. B. (2002). “The neurobiology of social affiliation and pair bonding,” in *Hormones, Brain, and Behavior* eds Pfaff D. Arnold A. M. Etgen A. M. Fahrbach S. E. Rubin R. T. (San Diego, CA: Academic Press) 299–337.

[B43] Casasanto D. Lupyan G. (2015). “All concepts are ad hoc concepts,” in *The Conceptual Mind: New Directions in the Study of Concepts* eds Margolis E. Laurence S. (Cambridge: MIT Press) 543–566.

[B44] Chaves V. E. Tilelli C. Q. Brito N. A. Brito M. N. (2013). Role of oxytocin in energy metabolism. *Peptides* 45 9–14. 10.1016/j.peptides.2013.04.010 23628372

[B45] Chemero A. (2009). *Radical Embodied Cognitive Science*. Cambridge, MA MIT Press.

[B46] Cho M. DeVries A. Williams J. Carter C. (1999). The effects of oxytocin and vasopressin on partner preference in male and female prairie voles. *Behav. Neurosci.* 113 1071–1079. 10.1037/0735-7044.113.5.1071 10571489

[B47] Clark A. (1998). *Being there: Putting the Brain, Body, and World Together Again*. Cambridge, MA: MIT Press.

[B48] Coan J. A. Allen J. J. B. (2003). Frontal EEG activity and the behavioral activation and inhibition systems. *Psychophysiology* 40 106–114. 10.1111/1469-8986.00011 12751808

[B49] Coan J. A. Beckes L. Allen J. P. (2013). Childhood maternal support and neighborhood quality moderate the social regulation of neural threat responding in adulthood. *Int. J. Psychophysiol.* 88 224–231. 10.1016/j.ijpsycho.2013.04.006 23639347 PMC3726257

[B50] Coan J. A. Schaefer H. S. Davidson R. J. (2006). Lending a hand: social regulation of the neural response to threat. *Psychol. Sci.* 17 1032–1039. 10.1111/j.1467-9280.2006.01832.x 17201784

[B51] Cohen D. Leung A. K.-Y. (2009). The hard embodiment of culture. *Eur. J. Soc. Psychol.* 39 1278–1289. 10.1002/ejsp.671

[B52] Cohen S. Janicki-Deverts D. (2009). Can we improve our physical health by altering our social networks? *Perspect. Psychol. Sci.* 4 375–378. 10.1111/j.1745-6924.2009.01141.x 20161087 PMC2744289

[B53] Craik K. J. W. (1943). *The Nature of Explanation*. Cambridge: Cambridge University Press.

[B54] Damasio A. (1999). *The Feeling of What Happens: Body and Emotion in the Making of Consciousness*. Orlando, FL: Harcourt.

[B55] Dunbar R. I. M. Shultz S. (2007). Evolution in the social brain. *Science* 317 1344–1347. 10.1126/science.1145463 17823343

[B56] Ebersole C. R. Atherton O. E. Belanger A. L. Skulborstad H. M. Adams R. B. Allen J. (2015). *Many Labs 3: Evaluating ParticipantPool Quality Across the Academic Semester via Replication*. Available at: osf.io/ct89g

[B57] Eriksson M. Björkstrand E. Smedh U. Alster P. Matthiesen A.-S. Uvnäs-Moberg K. (1994). Role of vagal nerve activity during suckling. Effects on plasma levels of oxytocin, prolactin, VIP, somatostatin, insulin, glucagon, glucose and of milk secretion in lactating rats. *Acta Physiol. Scand.* 151 453–459. 10.1111/j.1748-1716.1994.tb09767.x 7976418

[B58] Fiske A. P. (2004). “Relational models theory 2.0” in *Relational Models Theory: A Contemporary Overview* ed. Haslam N. (London: Lawrence Erlbaum Publishers) 3–25.

[B59] Fiske A. P. (2012). Metarelational models: configurations of social relationships. *Eur. J. Soc. Psychol.* 42 2–18. 10.1002/ejsp.847

[B60] Fodor J. Pylyshyn Z. (1988). Connectionism and the cognitive architecture. *Cognition* 28 3–71. 10.1016/0010-0277(88)90031-5 2450716

[B61] Fransson A. Karlsson H. Nilsson K. (2005). Temperature variation in newborn babies: importance of physical contact with the mother. *Arch. Dis. Child. Fetal Neonatal Ed.* 90 F500–F504. 10.1136/adc.2004.066589 16244210 PMC1721966

[B62] Gallagher E. N. Vella-Brodick D. A. (2008). Social support and emotional intelligence as predictors of subjective well-being. *Pers. Individ. Differ.* 44 1551–1561. 10.1016/j.paid.2008.01.011

[B63] Ghorbani N. Watson P. J. Krauss S. W. Davison H. K. Bing M. N. (2004). Private self-consciousness factors: relationships with need for cognition, locus of control, and obsessive thinking in Iran and the United States. *J. Soc. Psychol.* 144 359–372. 10.3200/SOCP.144.4.359-372 15279327

[B64] Gibson J. J. (1979). *The Ecological Approach to Visual Perception*. Boston, MA: Houghton-Miﬄin.

[B65] Gorzalka B. B. Lester G. L. L. (1987). Oxytocin-induced facilitation of lordosis behaviour is progesterone-dependent. *Neuropeptides* 10 55–65. 10.1016/0143-4179(87)90089-8 3670568

[B66] Gracanin A. Bylsma L. M. Vingerhoaets J. J. M. (2014). Is crying a self-soothing behavior. *Front. Psychol.* 5:502 10.3389/fpsyg.2014.00502 PMC4035568 24904511

[B67] Gregory S. G. Connelly J. J. Towers A. J. Johnson J. Biscocho D. Markunas C. A. (2009). Genomic and epigenetic evidence for oxytocin receptor deficiency in autism. *BMC Med.* 7:62 10.1186/1741-7015-7-62 PMC2774338 19845972

[B68] Griffiths P. E. Scarantino A. (2009). “Emotions in the wild: the situated perspective on emotion,” in *Cambridge Handbook of Situated Cognition* eds Robbins P. Aydede M. (Cambridge: Cambridge University Press) 437–453. 10.1017/CBO9780511816826.023

[B69] Grillon C. Krimsky M. Charney D. R. Vytal K. Ernst M. Cornwell B. (2013). Oxytocin increases anxiety to unpredictable threat. *Mol. Psychiatry* 18 958–960. 10.1038/mp.2012.156 23147382 PMC3930442

[B70] Grippo A. J. Trahanas D. M. Zimmerman R. R. II Porges S. W. Carter C. S. (2009). Oxytocin protects against negative behavioral and autonomic consequences of long-term social isolation. *Psychoneuroendocrinology* 34 1542–1553. 10.1016/j.psyneuen.2009.05.017 19553027 PMC2841348

[B71] Gross E. B. Proffitt D. R. (2013). The economy of social resources and its influence on social perceptions. *Front. Hum. Neurosci.* 7:772 10.3389/fnhum.2013.00772 PMC3832788 24312039

[B72] Harvey A. T. Hennessy M. B. (1995). Corticotropin-releasing factor modulation of the ultrasonic vocalization rate of isolated rat pups. *Dev. Brain Res.* 87 125–134. 10.1016/0165-3806(95)00064-K 7586494

[B73] Hasher L. Zacks R. T. (1979). Automatic and effortful processes in memory. *J. Exp. Psychol. Gen.* 108 356–388. 10.1037/0096-3445.108.3.356

[B74] Hazan C. Shaver P. (1987). Romantic love conceptualized as an attachment process. *J. Pers. Soc. Psychol.* 5 511–524. 10.1037/0022-3514.52.3.511 3572722

[B75] Hazan C. Shaver P. (1992). “Broken attachments,” in *Close Relationship Loss: Theoretical Approaches* ed. Orbuch T. L. (Hillsdale, NJ: Lawrence Erlbaum Associated Inc.) 90–108.

[B76] Hendriks-Jansen H. (1996). *Catching Ourselves in the Act*. Cambridge, MA: MIT Press.

[B77] Herman B. H. Panksepp J. (1978). Effects of morphine and naloxone on social attachment in infant guinea pigs. *Pharmacol. Biochem. Behav.* 9 213–220. 10.1016/0091-3057(78)90167-3 568801

[B78] Herman B. H. Panksepp J. (1981). Ascending endorphin inhibition of distress vocalization. *Science* 211 1060–1062. 10.1126/science.7466377 7466377

[B79] Hofer M. A. (1984). Relationships as regulators: a psychobiologic perspective on bereavement. *Psychosom. Med.* 46 183–197. 10.1097/00006842-198405000-00001 6739679

[B80] Hofer M. A. (1987). Early social relationships: a psychobiologist’s view. *Child Dev.* 58 633–647. 10.2307/1130203 3608643

[B81] Hofer M. A. (2006). Psychobiological roots of early attachment. *Curr. Dir. Psychol. Sci.* 15 84–88. 10.1111/j.0963-7214.2006.00412.x

[B82] Holt-Lunstad J. Smith T. B. Layton J. B. (2010). Social relationships and mortality risk: a meta-analytic review. *PLOS Med.* 7:e1000316 10.1371/journal.pmed.1000316 PMC2910600 20668659

[B83] IJzerman H. Cohen D. (2011). Grounding cultural syndroms: body comportment and values in Honor and Dignity cultures. *Eur. J. Soc. Psychol.* 41 456–467. 10.1002/ejsp.806

[B84] IJzerman H. Gallucci M. Pouw W. T. J. L. Weissgerber C. S. Van Doesum N. J. Williams K. D. (2012). Cold-blood loneliness: social exclusion leads to lower skin temperatures. *Acta Psychol.* 140 283–288. 10.1016/j.actpsy.2012.05.002 22717422

[B85] IJzerman H. Janssen J. Coan J. A. (2015a). Maintaining warm, trusting relationships with brands: increased temperature perceptions after thinking of communal bonds. *PLoS ONE* 10:e0125194 10.1371/journal.pone.0125194 PMC4411151 25915686

[B86] IJzerman H. Coan J. A. Wagemans F. A. M. Missler M. A. Van Beest I. Lindenberg S. M. (2015b). *A Theory of Social Thermoregulation in Human Primates*. Available at: http://papers.ssrn.com/sol3/papers.cfm?abstract_id=2521717 10.3389/fpsyg.2015.00464 PMC4404741 25954223

[B87] IJzerman H. Koole S. L. (2011). From perceptual rags to metaphoric riches: bodily, social, and cultural constraints on socio-cognitive metaphors. *Psychol. Bull.* 137 355–361. 10.1037/a0022373 21355634

[B88] IJzerman H. Semin G. R. (2009). The thermometer of social relations: mapping social proximity on temperature. *Psychol. Sci.* 20 1214–1220. 10.1111/j.1467-9280.2009.02434.x 19732385

[B89] IJzerman H. Semin G. R. (2010). Temperature perceptions as a ground for social proximity. *J. Exp. Soc. Psychol.* 46 867–873. 10.1016/j.jesp.2010.07.015

[B90] Israel S. Lerer E. Shalev I. Uzefovsky F. Riebold M. Laiba E. (2009). The oxytocin receptor (OXTR) contributes to prosocial fund allocations in the dictator game and the social value orientations task. *PLoS ONE* 4:e5535 10.1371/journal.pone.0005535 PMC2680041 19461999

[B91] Jack A. Connelly J. J. Morris J. P. (2012). DNA methylation of the oxytocin receptor gene predicts neural response to ambiguous social stimuli. *Front. Hum. Neurosci.* 6:280 10.3389/fnhum.2012.00280 PMC3467966 23087634

[B92] James W. (1884). What is an emotion? *Mind* 9 188–205. 10.1093/mind/os-IX.34.188

[B93] Johnson S. M. Burgess Moser M. Beckes L. Smith A. Dagleish T. Halchuk R. (2013). Soothing the threatened brain: leveraging contact comfort with emotionally focused therapy. *PLoS ONE* 8:e79314 10.1371/journal.pone.0079314 PMC3835900 24278126

[B94] Kalin N. H. Shelton S. E. Barksdale C. M. (1988). Opiate modulation of separation-induced distress in non-human primates. *Brain Res.* 440 285–292. 10.1016/0006-8993(88)90997-3 3359215

[B95] Kaplan H. S. Gangestad S. W. (2005). “Life history theory and evolutionary psychology,” in *The Handbook of Evolutionary Psychology* ed. Buss D. M. (Hoboken, NJ: Wiley) 68–95.

[B96] Kasahara Y. Sato K. Takayanagi Y. Mizukami H. Ozawa K. Hidema S. (2013). Oxytocin receptor in the hypothalamus is sufficient to rescue normal thermoregulatory function in male oxytocin receptor knockout mice. *Endocrinology* 154 4305–4315. 10.1210/en.2012-2206 24002032

[B97] Kasahara Y. Takayanagi Y. Kawada T. Itoi K. Nishimori K. (2007). Impaired thermoregulatory ability of oxytocin-deficient mice during cold-exposure. *Biosci. Biotechnol. Biochem.* 71 3122–3126. 10.1271/bbb.70498 18071238

[B98] Keverne E. B. Levy F. Poindron P. Lindsay D. R. (1983). Vaginal stimulation: an important determinant of bonding in sheep. *Science* 219 81–83. 10.1126/science.6849123 6849123

[B99] Keverne E. B. Martensz N. D. Tuite B. (1989). Beta-endorphin concentrations in cerebrospinal fluid of monkeys influenced by grooming relationships. *Psychoneuroendocrinology* 14 155–161. 10.1016/0306-4530(89)90065-6 2525263

[B100] Koole S. L. Tops M. Strübin S. Bouw J. Schneider I. K. Jostmann N. B. (2014). “The ego fixation hypothesis: involuntary persistence of self-control,” in *The Control within: Motivation and its Regulation* eds Forgas J. P. Harmon-Jones E. (New York, NY: Psychology Press) 95–112.

[B101] Kosfeld M. Heinrichs M. Zak P. J. Fischbacher U. Fehr E. (2005). Oxytocin increases trust in humans. *Nature* 435 673–676. 10.1038/nature03701 15931222

[B102] Kuhn C. M. Pauk J. Schanberg S. M. (1990). Endocrine responses to mother-infant separation in developing rats. *Dev. Psychobiol.* 23 395–410. 10.1002/dev.420230503 2253817

[B103] Kumsta R. Hummel E. Chen F. S. Heinrichs M. (2013). Epigenetic regulation of the oxytocin receptor: implications for behavioral neuroscience. *Front. Neuroendocrine Sci.* 7:83 10.3389/fnins.2013.00083 PMC3661945 23734094

[B104] Lakoff G. Johnson M. (1999). *Philosophy in the Flesh*. Arizona, AZ: Basic Books.

[B105] Landau M. J. Meier B. P. Keefer L. A. (2010). A metaphor-enriched social cognition. *Psychol. Bull.* 136 1045–1067. 10.1037/a0020970 20822208

[B106] Lee S. W. S. Schwarz N. (2012). Bidirectionality, mediation, and moderation of metaphorical effects: the embodiment of social suspicion and fishy smells. *J. Pers. Soc. Psychol.* 103 737–749. 10.1037/a0029708 22905770

[B107] Levine A. S. Morley J. E. Gosnell B. A. Billington C. J. Bartness T. J. (1985). Opioids and consummatory behavior. *Brain Res. Bull.* 14 663–672. 10.1016/0361-9230(85)90116-9 2992720

[B108] Lucht M. Barnow S. Sonnenfeld C. Rosenberger A. Grabe H. J. Schroeder W. (2009). Associations between the oxytocin receptor gene (OXTR) and affect, loneliness, and intelligence in normal subjects. *Prog. Neuropsychopharmacol. Biol. Psychiatry* 33 860–866. 10.1016/j.pnpbp.2009.04.004 19376182

[B109] MacArthur R. H. Wilson E. O. (1967). *The Theory of Island Biogeography*. Princeton, NJ: Princeton University Press.

[B110] Mandler J. M. (2004). A summary of the foundations of mind: origins of conceptual thought. *Dev. Sci.* 7 499–505. 10.1111/j.1467-7687.2004.00369.x

[B111] Marsh K. L. Richardson M. J. Baron R. M. Schmidt R. C. (2006). Contrasting approaches to perceiving and acting with others. *Ecol. Psychol.* 18 1–37. 10.1207/s15326969eco1801_1

[B112] Marsh K. L. Richardson M. J. Schmidt R. C. (2009). Social connection through joint action and interpersonal coordination. *Top. Cogn. Sci.* 1 320–339. 10.1111/j.1756-8765.2009.01022.x 25164936

[B113] McGowan P. O. Sasaki A. D’Alessio A. C. Dymov S. Labonte B. Szyf M. (2009). Epigenetic regulation of the glucocorticoid receptor in human brain associates with childhood abuse. *Nat. Neurosci.* 12 342–348. 10.1038/nn.2270 19234457 PMC2944040

[B114] Mikulincer M. (1995). Attachment style and the mental representation of the self. *J. Pers. Soc. Psychol.* 69 1203–1215. 10.1037/0022-3514.69.6.1203

[B115] Mikulincer M. Shaver P. R. (2003). “The attachment behavioral system in adulthood: activation, psychodynamics, and interpersonal processes,” in *Advances in Experimental Social Psychology* Vol. 35 ed. Zanna M. P. (New York, NY: Academic Press) 53–152.

[B116] Mineka S. Suomi S. J. (1978). Social separation in monkeys. *Psychol. Bull.* 85 1376–1400. 10.1037/0033-2909.85.6.1376 104324

[B117] Moore-Ede M. C. (1986). Physiology of the circadian timing system: predictive versus reactive homeostasis. *Am. J. Physiol.* 250 R737–R752. 10.1155/2015/825802 3706563

[B118] Nelson E. E. Panksepp J. (1998). Brain substrates of infant-mother attachment: contributions of opioids, oxytocin, and norepinephrine. *Neurosci. Biobehav. Rev.* 22 437–452. 10.1016/S0149-7634(97)00052-3 9579331

[B119] Oishi S. Schiller J. Gross E. B. (2013). Felt understanding and misunderstanding affect the perception of pain, slant, and distance. *Soc. Psychol. Pers. Sci.* 4 259–266. 10.1177/1948550612453469

[B120] Panksepp J. (1998). *Affective Neuroscience: The Foundations of Human and Animal Emotions*. New York, NY: Oxford University Press.

[B121] Panksepp J. Najam N. Soares F. (1980a). Morphine reduces social cohesion in rats. *Pharmacol. Biochem. Behav.* 11 131–134. 10.1016/0091-3057(79)90002-9 504292

[B122] Panksepp J. Bean N. J. Bishop P. Vilberg T. Sahley T. L. (1980b). Opioid blockade and social comfort in chicks. *Pharmacol. Biochem. Behav.* 13 673–683. 10.1016/0091-3057(80)90011-8 7443737

[B123] Panksepp J. Siviy S. Normansell L. (1985). “Brain opioids and social emotion,” in *The Psychobiology of attachment and separation* eds Reite M. Fields T. (New York, NY: Academic Press) 3–49. 10.1016/B978-0-12-586780-1.50006-9

[B124] Pedersen C. A. Caldwell J. D. Walker C. Ayers G. Mason G. A. (1994). Oxytocin activates the postpartum onset of rat maternal behavior in the ventral tegmental and medial preoptic areas. *Behav. Neurosci.* 108 1163–1171. 10.1037/0735-7044.108.6.1163 7893408

[B125] Pedersen C. A. Prange A. J. (1979). Induction of maternal behavior in virgin rats after intracerebroventricular administration of oxytocin. *Proc. Nat. Acad. Sci.* 76 6661–6665. 10.1073/pnas.76.12.6661 293752 PMC411928

[B126] Piaget J. Inhelder B. (1969). *The Psychology of the Child*. New York, NY: Basic Books.

[B127] Pollatos O. Herbert B. M. Kaufmann C. Auer D. P. Schandry R. (2007). Interoceptive awareness, anxiety and cardiovascular reactivity to isometric exercise. *Int. J. Psychophysiol.* 65 167–173. 10.1016/j.ijpsycho.2007.03.005 17449123

[B128] Porges S. W. (2007). The polyvagal perspective. *Biol. Psychol.* 74 116–143. 10.1016/j.biopsycho.2006.06.009 17049418 PMC1868418

[B129] Porges S. W. (2011). *The Polyvagal Theory: Neurophysiological Foundations of Emotions, Attachment, Communication and Self-regulation*. New York, NY: WW Norton.

[B130] Proffitt D. R. (2006). Embodied perception and the economy of action. *Perspect. Psychol. Sci.* 1 110–122. 10.1111/j.1745-6916.2006.00008.x 26151466

[B131] Raichle M. E. MacLeod A. M. Snyder A. Z. Powers W. J. Gusnard D. A. Shulman G. L. (2001). Inaugural article: a default mode of brain function. *Proc. Nat. Acad. Sci.* 98 676–682. 10.1073/pnas.98.2.676 11209064 PMC14647

[B132] Raison C. L. Hale M. W. Williams L. E. Wager T. D. Lowry C. A. (2015). Somatic influences on subjective well-being and affective disorders: the convergence of thermosensory and central serotonergic systems. *Front. Psychol. Cogn.* 5:1580 10.3389/fpsyg.2014.01580 PMC4292224 25628593

[B133] Ripoll L. H. Snyder R. Steele H. Siever L. J. (2013). The neurobiology of empathy in borderline personality disorder. *Curr. Psychiatry Rep.* 15 344 10.1007/s11920-012-0344-1 23389774

[B134] Rodrigues S. M. Saslow L. R. Garcia N. John O. P. Keltner D. (2009). Oxytocin receptor genetic variation relates to empathy and stress reactivity in humans. *Proc. Natl. Acad. Sci. U.S.A.* 106 21437–21441. 10.1073/pnas.0909579106 19934046 PMC2795557

[B135] Rosenfeld P. Wetmore J. B. Levine S. (1992). Effects of repeated maternal separations of the adrenocortical response to stress of preweanling rats. *Physiol. Behav.* 52 787–791. 10.1016/0031-9384(92)90415-X 1409954

[B136] Sanders J. T. (1997). An ontology of affordances. *Ecol. Psychol.* 9 97–112. 10.1207/s15326969eco0901_4

[B137] Schnall S. Harber K. Stefanucci J. Proffitt D. R. (2008). Social support and the perception of geographical slant. *J. Exp. Soc. Psychol.* 44 1246–1255. 10.1016/j.jesp.2008.04.011 22389520 PMC3291107

[B138] Selye H. (1974). *Stress Without Distress*. Philadelphia, PA: J.B. Lippincott Company 171.

[B139] Shapiro S. Salas M. (1970). Behavioral responses of infant rats to maternal odor. *Physiol. Behav.* 5 815–817. 10.1016/0031-9384(70)90285-4 5522499

[B140] Simmons W. K. Rapuano K. M. Kallman S. J. Ingeholm J. E. Miller B. Gotts S. J. (2013). Category-specific integration of homeostatic signals in caudal but not rostral human insula. *Nat. Neurosci.* 16 1551–1552. 10.1038/nn.3535 24077565 PMC3835665

[B141] Simpson J. A. (2007). “Foundations of interpersonal trust,” in *Social Psychology: Handbook of Basic Principles* 2nd Edn eds Kruglanski A. W. Higgins E. T. (New York, NY: Guilford) 587–607.

[B142] Simpson J. A. Rholes W. S. (2012). “Adult attachment orientations, stress, and romantic relationships,” in *Advances in Experimental Social Psychology* Vol. 45 eds Devine P. G. Plant A. Olson J. Zanna M. (New York, NY: Elsevier) 279–328.

[B143] Simpson J. A. Rholes W. S. Nelligan J. S. (1992). Support seeking and support giving within couples in an anxiety-provoking situation: the role of attachment styles. *J. Pers. Soc. Psychol.* 62 434–446. 10.1037/0022-3514.62.3.434

[B144] Simpson J. A. Rholes W. S. Phillips D. (1996). Conflict in close relationships: an attachment perspective. *J. Pers. Soc. Psychol.* 71 899–914. 10.1037/0022-3514.71.5.899 8939040

[B145] Skinner B. F. (1953). *Science and Human Behavior*. Toronto, ON: Macmillan Company.

[B146] Smotherman W. P. Robinson S. R. (1987). Prenatal expression of species typical action patterns in the rat fetus (*Rattus norvegicus*). *J. Comp. Psychol.* 101 190–196. 10.1037/0735-7036.101.2.190 3608424

[B147] Srinivasan M. Carey S. (2010). The long and the short of it: on the nature and origin of functional overlap between representations of space and time. *Cognition* 116 217–241. 10.1016/j.cognition.2010.05.005 20537324 PMC2900540

[B148] Sroufe L. A. (2005). Attachment and development: a prospective, longitudinal study from birth to adulthood. *Attach. Hum. Dev.* 7 349–367. 10.1080/14616730500365928 16332580

[B149] Stanton M. E. Wallstrom J. Levine S. (1987). Maternal contact inhibits pituitary-adrenal stress responses in preweanling rats. *Dev. Psychobiol.* 20 131–145. 10.1002/dev.420200204 3582776

[B150] Stearns S. C. (1992). *The Evolution of Life Histories*. Oxford: Oxford University Press.

[B151] Stefanucci J. K. Proffitt D. R. Banton T. Epstein W. (2005). Distances appear different on hills. *Percept. Psychophys.* 67 1052–1060. 10.3758/BF03193631 16396013

[B152] Sullivan R. M. Hofer M. A. Brake S. C. (1986). Olfactory-guided orientation in neonatal rats is enhanced by a conditional change in behavior state. *Dev. Psychobiol.* 19 615–623. 10.1002/dev.420190612 3803729

[B153] Szymkow A. Chandler J. IJzerman H. Parzuchowski M. Wojciszke B. (2013). Warmer hearts, warmer rooms: focusing on communal but not agentic traits increases estimates of ambient room temperature. *Soc. Psychol.* 44 167–176. 10.1027/1864-9335/a000147

[B154] Takano K. Tanno Y. (2009). Self-rumination, self-reflection, and depression: self- rumination counteracts the adaptive effect of self-reflection. *Behav. Res. Ther.* 47 260–264. 10.1016/j.brat.2008.12.008 19181307

[B155] Takayanagi Y. Kasahara Y. Onaka T. Takahashi N. Kawada T. Nishimori K. (2008). Oxytocin receptor-deficient mice developed late-onset obesity. *Neuroreport* 19 951–959. 10.1097/WNR.0b013e3283021ca9 18520999

[B156] Tops M. Boksem M. A. S. (2011). A potential role of the inferior frontal gyrus and AI in cognitive control, brain rhythms and event-related potentials. *Front. Psychol.* 2:330 10.3389/fpsyg.2011.00330 PMC3212750 22084637

[B157] Tops M. Boksem M. A. S. Luu P. Tucker D. M. (2010). Brain substrates of behavioral programs associated with self-regulation. *Front. Psychol.* 1:152 10.3389/fpsyg.2010.00152 PMC3157933 21887146

[B158] Tops M. Boksem M. S. A. Quirin M. IJzerman H. Koole S. L. (2014). *Internally-directed Cognition and Mindfulness: An Integrative Perspective Derived from Reactive Versus Predictive Control Systems Theory*. Available at: http://ssrn.com/abstract=2401659 (accessed February 26, 2014) 10.2139/ssrn.2401659 PMC4033157 24904455

[B159] Tost H. Kolachana B. Hakimi S. Lemaitre H. Verchinski B. A. Mattay V. S. (2010). A common allele in the oxytocin receptor gene (OXTR) impacts prosocial temperament and human hypothalamic-limbic structure and function. *Proc. Natl. Acad. Sci. U.S.A.* 107 13936–13941. 10.1073/pnas.1003296107 20647384 PMC2922278

[B160] Trapnell P. D. Campbell J. D. (1999). Private self-consciousness and the five-factor model of personality: distinguishing rumination from reflection. *J. Pers. Soc. Psychol.* 76 284–304. 10.1037/0022-3514.76.2.284 10074710

[B161] Trivers R. L. (1972). “Parental investment and sexual selection,” in *Sexual Selection and the Descent of Man* ed. Campbell B. (New York, NY: Aldine de Gruyter) 136–179.

[B162] Troisi A. Frazzetto G. Carola V. Di Lorenzo G. Coviello M. D’Amato F. R. (2010). Social hedonic capacity is associated with the A118G polymorphism of the mu-opioid receptor gene (OPRM1) in adult healthy volunteers and psychiatric patients. *Soc. Neurosci.* 6 88–97. 10.1080/17470919.2010.482786 20486014

[B163] Uvnas-Moberg K. Bruzelius G. Alster P. Lundeberg T. (1993). The antinociceptive effect of non-noxious sensory stimulation is mediated partly through oxytocinergic mechanisms. *Acta Physiol. Scand.* 149 199–204. 10.1111/j.1748-1716.1993.tb09612.x 8266809

[B164] Wakerley J. B. Lincoln D. W. (1973). The milk-ejection reflex of the rat: a 20- to 40-fold acceleration in the firing of paraventricular neurones during oxytocin release. *J. Endocrinol.* 57 477–493. 10.1677/joe.0.0570477 4577217

[B165] Walum H. Lichtenstein P. Neiderhiser J. M. Reiss D. Ganiban J. M. Spotts E. L. (2011). Variation in the oxytocin receptor gene is associated with pair-bonding and social behavior. *Biol. Psychiatry* 71 419–426. 10.1016/j.biopsych.2011.09.002 22015110 PMC3266986

[B166] Watson P. J. Morris R. J. Ramsey A. Hickman S. E. Waddell M. G. (1996). Further contrasts between self-reflectiveness and internal state awareness factors of private self- consciousness. *J. Psychol. Interdiscip. Appl.* 130 183–192. 10.1080/00223980.1996.9915000 8636907

[B167] Waytz A. Mitchell J. P. (2011). Two mechanisms for simulating others minds: dissociations between mirroring and self-projection. *Curr. Dir. Psychol. Sci.* 20 197–200. 10.1177/0963721411409007

[B168] Whalen P. J. (2007). The uncertainty of it all. *Trends Cogn. Sci.* 11 499–500. 10.1016/j.tics.2007.08.016 18024182

[B169] Williams L. E. Bargh J. A. (2008). Experiencing physical warmth promotes interpersonal warmth. *Science* 322 606–607. 10.1126/science.1162548 18948544 PMC2737341

[B170] Williams L. E. Huang J. Y. Bargh J. A. (2009). The scaffolded mind: higher mental processes are grounded in early experience of the physical world. *Eur. J. Soc. Psychol.* 39 1257–1267. 10.1002/ejsp.665 20046813 PMC2799930

[B171] Wilson A. D. Golonka S. (2013). Embodied cognition is not what you think it is. *Front. Psychol.* 4:58 10.3389/fpsyg.2013.00058 PMC3569617 23408669

[B172] Winslow J. T. Insel T. R. (1993). Effects of central vasopressin administration to infant rats. *Eur. J. Pharmacol.* 233 101–107. 10.1016/0014-2999(93)90354-K 8472738

[B173] Witt D. M. Carter C. S. Walton D. (1990). Central and peripheral effects of oxytocin administration in prairie voles (*Microtus ochrogaster*). *Pharmacol. Biochem. Behav.* 37 63–69. 10.1016/0091-3057(90)90042-G 2263668

[B174] Witt D. M. Insel T. R. (1991). A selective oxytocin antagonist attenuates progesterone facilitation of female sexual behavior. *Endocrinology* 128 3269–3276. 10.1210/endo-128-6-3269 1645266

[B175] Zajonc R. B. Markus H. (1984). “Affect and cognition: the hard interface,” in *Emotions, Cognitions, and Behavior* eds Izard C. Kagan J. Zajonc R. B. (Cambridge: Cambridge University Press) 73–102.

[B176] Zak P. J. Stanton A. A. Ahmadi S. (2007). Oxytocin increases generosity in humans. *PLoS ONE* 2:e1128 10.1371/journal.pone.0001128 PMC2040517 17987115

[B177] Zeifman D. Hazan C. (2008). “Attachment: pair bonds as attachments: re-evaluating the evidence,” in *Handbook of Attachment: Theory, Research, and Clinical Applications* 2nd Edn eds Cassidy J. Shaver P. R. (New York, NY: Guilford Press) 436–455.

[B178] Zhong C. Leonardelli G. J. (2008). Cold and lonely: does social exclusion feel literally cold? *Psychol. Sci.* 19 838–842. 10.1111/j.1467-9280.2008.02165.x 18947346

